# Assessing the Genetic Landscape of Animal Behavior

**DOI:** 10.1534/genetics.118.300712

**Published:** 2018-03-21

**Authors:** Ryan A. York

**Affiliations:** Department of Biology, Stanford University, California 94305

**Keywords:** behavior genetics, quantitative trait loci, GWAS, behavioral evolution

## Abstract

Although most animal behaviors are associated with some form of heritable genetic variation, we do not yet understand how genes sculpt behavior across evolution, either directly or indirectly. To address this, I here compile a data set comprised of over 1000 genomic loci representing a spectrum of behavioral variation across animal taxa. Comparative analyses reveal that courtship and feeding behaviors are associated with genomic regions of significantly greater effect than other traits, on average threefold greater than other behaviors. Investigations of whole-genome sequencing and phenotypic data for 87 behavioral traits from the *Drosophila* Genetics Reference Panel indicate that courtship and feeding behaviors have significantly greater genetic contributions and that, in general, behavioral traits overlap little in individual base pairs but increasingly interact at the levels of genes and traits. These results provide evidence that different types of behavior are associated with variable genetic bases and suggest that, across animal evolution, the genetic landscape of behavior is more rugged, yet predictable, than previously thought.

## Abstract (precis)

Recent years have seen an increase in studies that associate genomic loci with behavioral variation both within and across animal species. Ryan York compiles and analyzes over 1,000 of these loci, finding that the genetic...

NEARLY all behaviors are associated with some form of heritable genetic variation (Kendler and Greenspan 2006). This interplay between genetic and other forces that shape behavior is complex, and disentangling it occupies an array of research endeavors spanning disciplines from evolutionary biology to psychiatry. Accordingly, recent years have seen reasonable progress toward understanding the genetic architecture of certain behavioral traits using model systems (Reaume and Sokolowski 2011). The general conclusion from this research in mice, flies, worms, and humans is that the genetic architectures of behaviors generally fit an exponential distribution, with a small number of loci of moderate-to-large effect and a larger number of loci with small effects (Robertson 1967; Flint and Mackay 2009). However, owing to limits in data and methods, the extent to which genetic architectures vary across a full spectrum of behaviors and animal taxa has remained largely unexplored.

Behaviors can exhibit considerable variation in genetic influence. Comparative analyses reveal that behaviors vary substantially in heritability estimates, most often ranging between 10 and 50% (Mousseau and Roff 1987; Meffert *et al.* 2002; Kendler and Greenspan 2006). Analyses of individual behaviors reveal even greater diversity. For example, a single retroelement is responsible for variation in a courtship song between *Drosophila* species (Ding *et al.* 2016), while other traits may be associated with more complex or polygenic architectures (Flint and Mackay 2009). Furthermore, the structure and effect of genetic architectures may vary with behavioral traits, as suggested by the preponderance of large-effect loci found for insect courtship traits across multiple species (Arbuthnott 2009). Despite these observations, the extent to which behavioral traits may systematically vary across species and behaviors remains unknown. Understanding this could provide insights into how behaviors respond to evolutionary processes, the prospects for finding general principles in the genetic evolution of behavior, and even potentially why there has been such variable success in the mapping of human neuropsychiatric traits.

Here, using reports associating behavioral variation with the genes for specific traits across diverse species, I assemble a comparative behavior genetics resource composed of 1007 significant genomic loci from 114 QTL studies conducted in 30 species across five taxonomic classes. These data exploit the wealth of QTL mapping efforts that have worked to identify genomic regions associated with behavioral variation over the past several decades (Lander and Botstein 1989; Flint and Mackay 2009). With the compiled data set I in File S2 compare the genetic architecture of behavioral types across animal taxa. I then corroborate these observations and assay genetic processes involved in the early stages of behavioral differentiation in a natural population using whole-genome data from the *Drosophila* Genetic Resource Panel (DGRP). These analyses provide insight into the genetic architecture of behavior across animals and the interplay between specific behavioral traits and their genetic influence through evolutionary history.

## Materials and Methods

### QTL collection

I first identified behavioral QTL through a literature search querying online engines (*e.g.*, PUBMED) with the keywords “QTL,” “behavior,” “quantitative trait locus,” and “behavioral.” I analyzed the results and collected QTL for each relevant publication identified. To gather as many relevant QTL as possible over time, I expanded the search to include more specific terms relating to behaviors and categories of interest and to those referenced in previously identified papers. I filtered for loci reported as significant by the original authors, resulting in 1007 QTL from 115 studies. For each locus, I recorded the reported effect size (percent phenotypic variation explained), significance measure, genomic location, sample size, and the number of loci reported overall. QTL studies often report other measures in addition to those that I collected (*e.g.*, broad- or narrow-sense heritability). While it would be desirable to compare some of these across behaviors and taxonomic groups, I found that, within the studies assayed, the reporting of measures other than those I collected was very inconsistent and allowed for only extremely restricted comparisons. Since the measure used to report significance varied across studies, I converted all LOD scores to Log *P*-values in R (R Development Core Team 2015).

I next classified behaviors following the six groups used in the meta-analysis of mouse QTL studies done by Flint (2003). Several categories represented in our data set were not assayed in this original study (*e.g.*, courtship). In our classification of these, I attempted to strike a balance between breadth (to increase the tractability of our comparisons) and biological specificity. To do so, I required that a category be represented in at least two species or populations and that the classification match either that reported by the original authors or a reasonable division as reported by the animal behavior literature. The classification of a range of biological traits into broader categories is, of course, difficult and can repeatedly tempt debate; accordingly, this is discussed at length in Flint (2003). I offer that it is important to rigorously test results implicating a broadly defined category as interesting through comparisons of that category to the overall distribution of effects, with the goal of controlling for bias introduced by the original classifications (as is discussed below). All QTL and the associated measures mentioned here are available in data set S1.

### Phylogeny

**Figure 1:**
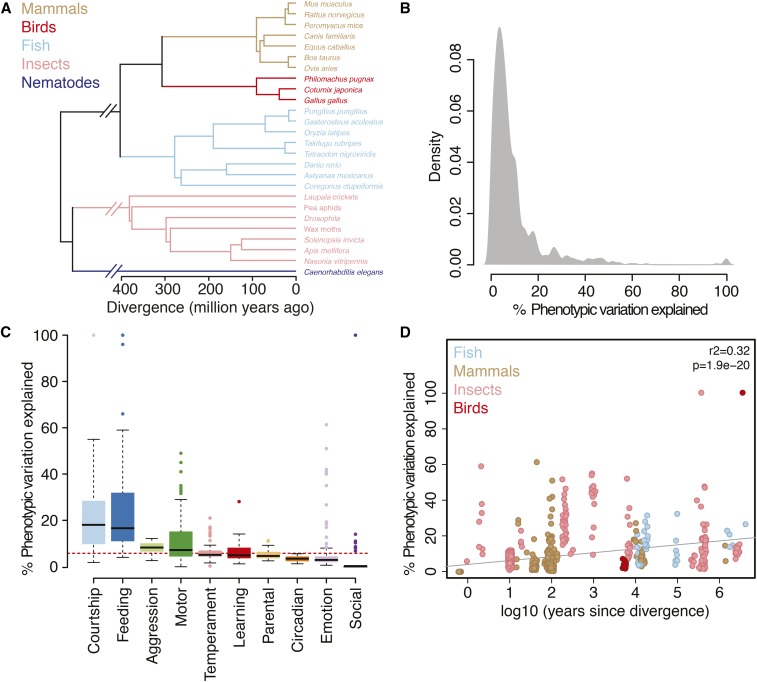
The genomic landscape of animal behavior. (A) Phylogeny of all species studied in which genomic loci were collected for the meta-analysis. (B) Density plot of the distribution of effect sizes for all behavioral traits studied. (C) Boxplot of effect sizes (% variation explained) by behavioral category. (D) Scatterplot of the relationship between evolutionary divergence (represented by the log_10_ of years since divergence) and effect size.

I used the phylogenetic relationships reported in Ponting (2008) as a template for the phylogeny of species examined (Figure 1A). I added unrepresented species and adjusted dates of evolutionary divergence using the most recent reports available for each specific clade/species. The following sources were used (along with the associated phylogenetic divergences):

Ruff/quail and chicken: Jarvis *et al.* (2014).
Quail and chicken: Kayang *et al.* (2006).
Nine-spined and three-spined stickleback: Guo *et al.* (2013).
Stickleback and teleost: Pfister *et al.* (2007).
Cave fish and teleost divergence: Briggs (2005).
Laupala cricket and insect divergence: Misof *et al.* (2014).
Wax moth and insects: Misof *et al.* (2014).
Pea aphids and insects: Misof *et al.* (2014).
*Peromyscus* and mice/rats: Bedford and Hoekstra (2015).
Solenopsis and *Apis*: Ward (2014).
Sheep and cows: Bibi (2013).
White fish and teleosts: Betancur-R *et al.* (2013).

### Effect size comparisons

Bayesian mixed effect model analyses were run using the MCMCglmm package (Hadfield 2010) in R 3.3.2 with standard inverse γ priors for random effects, a burn-in period of 10,000 iterations, and a total of 300,000 iterations thinned by 10. Model convergence was assessed by visual inspection of the output chains in addition to calculation of the Geldman–Rubin statistic from the CODA package (Plummer *et al.* 2006) using five chains for each model. In total, seven models comprising all combinations of the random effects were tested and then ranked based on the deviance information criterion (DIC) outputted by MCMCglmm. The results of this comparison are presented in Supplemental Material, Table S1 in File S1. The best-ranked model from these analyses was also tested using linear mixed effects regression in the lme4 package (Bates *et al.* 2015) in R. The model predicted effect size using behavioral category as a fixed effect and sample size and number of generations as random effects with random slopes and intercepts. The results from this model are presented in Table S2 in File S1.

Permutation tests of effect sizes were conducted in R using sampling without replacement. First, the observed mean for the behavioral category being tested was calculated, as was the number of measures for that trait included in the data set. The remaining effect sizes were then sampled 10,000 times. To produce a null distribution, mean effect sizes were computed for each permutation and stored. This null distribution was then used to calculated *P*-values for each category by comparing the number of times the null distribution was greater than the observed mean to the number of permutations. The results of this analysis are presented in Table S3 in File S1.

### Data collection of the DGRP lines

I downloaded the DGRP freeze 2.0 variant calls and plink files from the *Drosophila* genetics reference panel website (http://dgrp2.gnets.ncsu.edu). Raw data for phenotypic measures were downloaded from the following sources:

Starvation resistance: Mackay *et al.* (2012).
Startle response: Mackay *et al.* (2012).
Chill coma recovery time: Mackay *et al.* (2012).
Startle response under oxidative stress: Jordan *et al.* (2012).
Negative geotaxis under oxidative stress: Jordan *et al.* (2012).
Olfactory behavior (benzaldehyde): Swarup *et al.* (2013).
Courtship behavior: Gaertner *et al.* (2015).
Olfactory behavior (multiple measures): Arya *et al.* (2015).
Aggressive behavior: Shorter *et al.* (2015).
Food intake: Garlapow *et al.* (2015).
Alcohol sensitivity: Morozova *et al.* (2015).
Morphology: Vonesch *et al.* (2016).

I compiled the raw data into two tables for use in genome-wide analyses of SNP variation: one composed of the 87 behavioral traits obtained and another of the 26 morphological traits. For traits in which multiple measurements were reported I calculated the mean trait measurement and used this for subsequent analyses. I classified traits into behavioral categories in the same fashion as for the evolutionary QTL analyses.

### Heritability analyses

I first employed genome-wide complex trait analysis (GCTA) to survey genomic heritability across the 87 behavioral traits (Yang *et al.* 2011). For each trait, I used GREML v1.26.0 to obtain estimates of heritability from genome-wide SNP variation across all DGRP lines for which phenotypic measures were available. Using the plink files obtained from the DGRP website, I first created a genotype relatedness matrix for all DGRP lines with the flag–maf 0.01. Individual phenotype files (*.phen) were created for each trait, including fam and individual identifiers and the associate phenotypic measures for each DGRP line. I ran GREML for each phenotype separately. I then filtered for traits in which the reported *P*-value from GREML was < 0.05, resulting in 20 traits. Figure 3A shows the distribution of phenotypic variance explained by genome-wide SNPs as measured by the genotypic variance divided by phenotypic variance (Vg/Vp).

For the GCTA analyses of just genome-wide association studies (GWAS)-significant SNPs, I compiled a list of associated SNPs for each trait and built a separate genotype relatedness matrix for each by extracting just those SNPs from the plink bed files. I then reran GREML for each trait using the corresponding genotype relatedness matrix and testing only for the SNPs that it contained. As above, I then filtered for traits in which the reported *P*-value from GREML was < 0.05, resulting in 16 traits.

### Genome-wide association analyses

The plink and phenotype files from the GCTA analyses were used to conduct separate GWAS for each trait. I used plink v1.90 (Purcell *et al.* 2007) to conduct these tests on the combined phenotype matrix. Associations were then filtered for a *P*-value < 5 × 10^−6^. SNPs associated with multiple traits were identified and plotted using a binary heatmap with the heatmap2 function in R. Genes associated with multiple SNPs were identified using the variant annotation file available on the DGRP website.

I next assayed relationships between SNPs and multiple traits using the effect sizes (β) in the *.qassoc files outputted by plink. To do so, I compiled a matrix of the effect sizes for all traits at each of the 25,919 significant SNPs (Table S4). This matrix could then be directly queried for comparison of the effect sizes associated with a certain set of SNPs across traits of interest. To assess the overall structure of this data set, I used Spearman’s rank correlations to test the associations between all possible trait pairs. The results of this test were visualized using the clustering functionality of heatmap2 in R (Figure S3 in File S1).

### Tests for trait pair directionality

Directionality in the relationships between trait pairs was tested by first obtaining pairwise rank correlations for each trait pair in which both traits were associated with more than three significant SNPs (60 traits). For traits x and y, s_1_ is the vector of SNPs significantly associated with trait x and s_2_ is the vector of SNPs significantly associated with trait y. xx is the vector of effect sizes at s_1_ for trait x and xy is the vector of effect sizes at s_1_ for trait y. Similarly, yy is the vector of effect sizes at s_2_ for trait y and yx is the vector of effect sizes at s_2_ for trait x. Rank correlations can then be obtained for each in R:

x_cor= cor(xx,xy,method=“spearman”)

y_cor=cor(yy,yx,method=“spearman”)

Since the strongest signals of directionality would be cases in which the absolute value of x_cor-y_cor equals 1, I assessed directionality as a function of how close to 1 the absolute difference between the correlations was:

D= abs(1−abs(x_cor−y_cor))

I filtered for trait pairs in which ρ for one correlation was > 0.5 and for the other was < 0.1. I then tested the directional significance of each trait pair by permuting xx, xy, yy, and yx 1000 times and recomputed x_cor, y_cor, and D for each permutation. A *P*-value for each trait pair was calculated by comparing the vector of permuted D values (pseudo) to the observed D:

pseudo = c();

for (trial in 1:1000) {

pxx = sample(xx, length(xx), replace = F);

Bxy = sample(xy, length(xy), replace = F);

Byy = sample(yy, length(yy), replace = F);

Byx = sample(yx, length(yx), replace = F);

p_x_cor = cor(pxx, pxy, method=”spearman”, use=”pairwise.complete.obs”)

p_y_cor = cor(pyy, pyx, method=”spearman”, use=”pairwise.complete.obs”)

d = abs(p_x_cor-p_y_cor)

pD = abs(1-d)

pseudo[trial] = pD

}

p_value = sum(D > pD)/1000

The resulting *P*-values were adjusted using Bonferroni correction.

### Data availability

QTL data used for the meta-analysis are included File S2. DGRP GWAS and behavioral data are available through either the original publications or the DGRP website (http://dgrp2.gnets.ncsu.edu/).

## Results and Discussion

I performed a comprehensive analysis of results aggregated from 114 QTL studies conducted in 30 species across five taxonomic classes to assemble a comparative behavior genetics resource composed of 1007 significant genomic loci (File S2). The species examined represent over 500 million years of evolutionary divergence over a broad spectrum of phylogenetic data (Figure 1A). For each locus, I annotated the trait measured and its associated effect size (percent phenotypic variation explained), the reported measure of significance (*e.g.*, LOD score), genomic locus, and study sample size. I focused the analyses on the reported effect sizes to allow comparison of the genomic architecture of traits across studies similar to previous meta-analyses of behavioral QTL in mice and flies (Flint 2003; Flint and Mackay 2009).

I found that the distribution of effect sizes in the data set is similar to that found in these previous studies (Figure 1B). In the majority of loci (89.51%), the effect sizes are < 20% with a mean effect size of 9.54%, suggesting that the genetic bases of most behaviors assayed are complex and composed of many loci of moderate effect.

I next asked whether genetic architecture might vary across *types* of behavior. I identified 10 behavioral categories for which traits had been measured in at least two species (see supplemental methods in File S1). My null hypothesis was that individual categories would likely reflect the overall distribution seen across the data set, consistent with previous observations that QTL have relatively similar effect sizes across mouse and fly phenotypes (Flint 2003; Flint and Mackay 2009). Surprisingly, I found instead that behaviors differed significantly in their effect sizes. Specifically, loci associated with courtship (*n* = 124) explained significantly more phenotypic variance than all other behaviors combined (Kruskal–Wallis *P* = 6.7 × 10^−29^) and had a mean effect size three times larger than found in all other categories (Figure 1C). Loci associated with feeding behaviors (*n* = 11) also explained significantly more phenotypic variance than all other behaviors combined (*P* = 6.8 × 10^−13^), while emotion and social behaviors explained significantly less (*P* = 8.6 × 10^−33^; *P* = 2.5 × 10^−21^, respectively).

Given the heterogeneity of the experiments analyzed here, in addition to inborn issues of QTL mapping such as the Beavis effect (Beavis 1995), I next attempted to assess the extent to which experimental bias and artifacts may have contributed bias to these results. I first considered the effect of intraspecific (within species) compared to interspecific (between species) crosses used for the QTL mapping, a known source of influence in QTL studies (Broman 2001). Indeed, I found that experiments employing interspecific crosses identified loci of significantly higher effect (*P* = 4.5 × 10^−5^). To control for this quantitatively, I estimated phylogenetic divergence and generation times between the crosses used in each of the 115 studies (supplemental methods in File S1). There was a positive correlation between evolutionary divergence and effect size (*r*^2^ = 0.32, *P* = 1.9 × 10^−20^; Figure 1D and supplemental methods in File S1). I also considered sample size, a well-known source of bias for which there was a negative correlation with effect size (*r*^2^ = −0.37, *P* < 0.0001).

**Figure 2:**
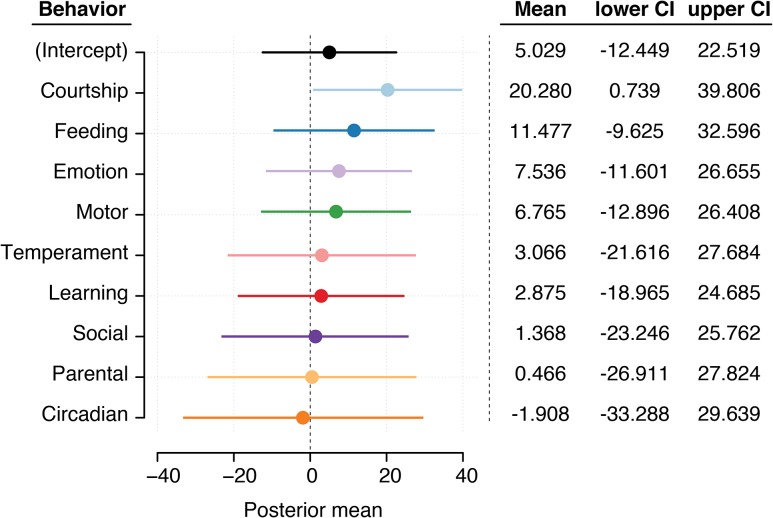
Results from Bayesian generalized linear mixed model on QTL effect sizes. Scatterplot of posterior means from the best fit MCMCg model (including generations diverged and sample size as random effects) with posterior means and 95% C.I. bounds. Colors correspond to behavioral category as in Figure 1.

In light of the potential confounding influences of sample size, evolutionary relationships, and inter- *vs.* intraspecific crossing schemes, I employed both Bayesian and frequentist mixed models to test the relationship between QTL effect sizes and behavioral traits. Using the R (R Development Core Team 2015) package MCMCglmm (Hadfield 2010), I compared the fit of multiple models incorporating sample size, evolutionary divergence (via generations), and inter- *vs.* intraspecific crossing schemes as random effects with behavioral trait as a fixed effect (supplemental methods and Table S1 in File S1). The best ranked of these Bayesian models used sample size and generation time as random effects (DIC = 4462.879). Notably, adding inter- *vs.* intraspecific crossing schemes as a random effect reduced the explanatory power of the model (Table S1 in File S1). Analyzing posterior means of the best fit model showed that courtship behavior had a significant influence on effect size (posterior mean = 20.280; 95% highest posterior density (HPD) = 0.739–39.806; Figure 2). Similarly, frequentist analysis of the same linear mixed effect model (incorporating sample size and generation time) using the lme4 package in R (Bates *et al.* 2015) demonstrated that courtship behavior had a significant influence on effect size (*b* = 20.131, SE = 5.985, *P* < 0.001) in addition to feeding (*b* = 13.721, SE = 4.494, *P* < 0.005; Table S2 in File S1).

Variation in the number of studies measuring a given behavioral trait was also explored as a possible confounding influence. For example, a specific behavior’s significance may be driven by a small number of studies with extremely large effect sizes. To explore this possibility, the observed mean effect sizes for each behavioral category were compared to null distributions obtained by permuting all other effect sizes 10,000 times without replacement. The mean effect sizes for courtship and feeding behaviors were both significantly greater than expected when compared to these permuted distributions (both traits: *P* < 0.0005). None of the other traits reached significance (Table S3 in File S1).

These results indicate that the genetic bases of courtship behaviors, and to a lesser extent feeding, significantly vary compared to other behaviors across taxa, suggesting that they may facilitate different responses to evolutionary pressures than other behavioral traits. Consistent with this notion, previous analyses of the QTL behavior literature in insects found that a majority of courtship traits are associated with few loci of particularly strong effect that play a potential role in rapid speciation through prezygotic isolation (Arbuthnott 2009). In addition, theoretical work has suggested that traits controlling local adaptation during speciation, such as courtship and feeding, evolve more rapidly if they are associated with a smaller number of loci (Gavrilets *et al.* 2007). Given the importance of behavior’s role in the early stages of speciation, it may be possible that, for the organisms and traits analyzed here, courtship and feeding traits with simpler genetic components of large effect were selected for during the evolution of these lineages. Given this, I next investigated to what the extent courtship and feeding behaviors may be associated with more heritable genetic architectures of greater effect when compared to other behavioral traits in a naturally interbreeding population.

To test this idea, I used the DGRP. The DGRP is comprised of over 200 inbred, fully sequenced *Drosophila melanogaster* lines isolated from a farmer’s market in Raleigh, North Carolina (Mackay *et al.* 2012). Phenotypic measures for a wide number of behavioral traits are available for the DGRP lines in addition to full genome sequence and variant information, making this resource unique in enabling us to ask larger-scale questions about variation and evolution in behavior. I collected phenotypic measures for 87 behavioral traits spanning eight categories, produced in nine separate GWAS (Jordan *et al.* 2012; Weber *et al.* 2012; Swarup *et al.* 2013; Arya *et al.* 2015; Gaertner *et al.* 2015; Garlapow *et al.* 2015; Morozova *et al.* 2015; Shorter *et al.* 2015).

**Figure 3:**
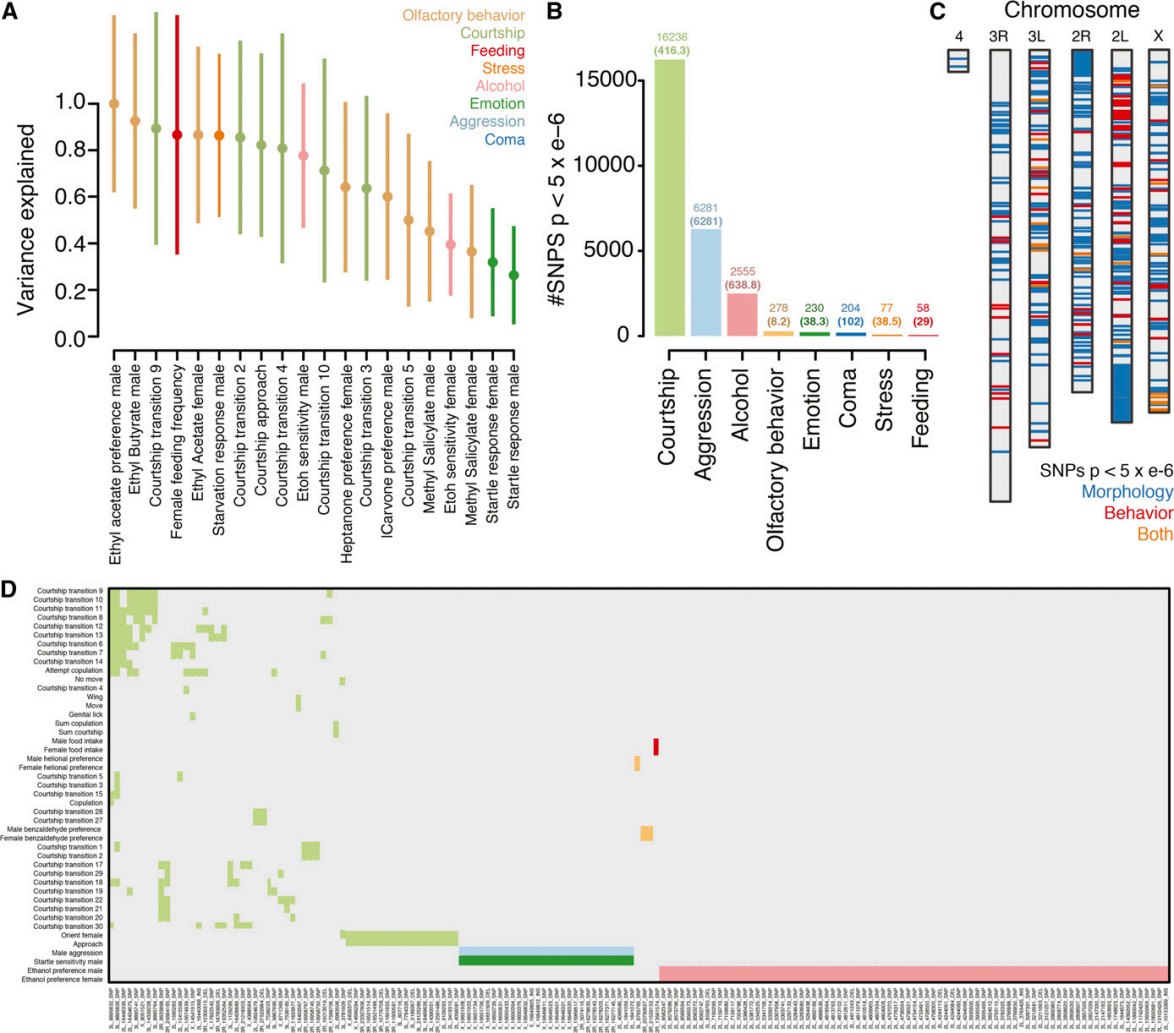
Comparative genome-wide analyses of the *Drosophila* Genetic Resource Panel. (A) Heritability estimates (genotypic variance divided by phenotypic variance) from genome-wide complex trait analysis for the 20 measures identified as significant (*P*-value < 0.05), colored by behavioral category. (B) Barplot summarizing the number of SNPs with *P* < 5 × 10^−6^ collected for each behavioral category from genome-wide association studies on 87 traits. The total number of SNPs for each trait are presented above each bar. The parenthesized numbers represent the total number of SNPs divided by the number of traits tested in that category. (C) The distribution of SNPs with *P* < 5 × 10^−6^ across the *D. melanogaster* genome for morphological (blue) and behavioral (red) traits and SNPs that associate with measures of both (orange). (D) Heatmap representing the distribution of shared SNPs with *P* < 5 × 10^−6^ across all behavioral traits. Plotted are SNPs that possess associations with at least two behavioral traits, colored by the categories highlighted in (A).

I first used GCTA to survey the extent to which the 87 behavioral traits varied in genomic heritability attributable to all autosomal SNPs (Yang *et al.* 2011). After running GCTA, 20 behavioral traits passed a *P*-value threshold of 0.05, indicating that autosomal SNPs could explain a significant amount of variation in these traits (Figure 3A and supplemental methods in File S1). The majority of these traits were enriched for involvement in courtship and feeding: 30% (6/20) were associated with courtship and 50% (10/20) were either involved in olfactory behavior or feeding. Notably, for a number of these traits, the vast majority of phenotypic variation could be explained by genome-wide SNPs, including preference for the food odorant ethyl acetate (99.99 ± 38.05%) and courtship transition 9 (89.38 ± 50.03%).

The previous analyses of QTL effect sizes suggested that the genomic architectures of courtship and feeding traits may be simpler and of higher effect. To test whether or not this was the case among the DGRP lines, I performed a separate GWAS for each trait across all lines with available phenotypic data and filtered for SNPs with a nominal *P*-value of 5 × 10^−6^ (supplemental methods in File S1). At this threshold, I found 25,919 SNPs (Figure 3B and Table S4). Recent studies have demonstrated the susceptibility of moderately sized next-generation mapping panels such as the DGRP to effect size overinflation via the Beavis effect (King and Long 2017), thus causing concern regarding the potential overestimation of effects, especially if some studies or traits show strong effects owing from consistently small sample sizes. Generally, though, I found a very weak relationship between the number of individuals genotyped and the GWAS *P*-value for each SNP (Spearman’s ρ = 0.0489; *P*-value *=* 3.296 × 10^−15^). This suggests that, while the Beavis effect is likely present given the inborn issues with genetic panels like the DGRP, the overall effect may be distributed across the studies and should be taken as a caveat when interpreting observed patterns in the data.

I reran GCTA for each trait using only SNPs identified at *P* < 5 × 10^−6^ from the GWAS (supplemental methods in File S1). This test is more conservative compared to genome-wide GCTA since it uses just the fraction of genomic variants that are significantly associated with each individual trait. After GCTA, I found 16 behavioral traits that passed the *P*-value threshold of *P* < 0.05. Half of these significant traits were courtship behaviors, including the top four traits with the most variation explained by GWAS SNPs (Figure S1 in File S1). The number of GWAS-significant SNPs for these 16 traits varied substantially and was positively correlated with the amount of phenotypic variance explained (Figure S1 in File S1). For traits with more SNPs, significant portions of the variance could be accounted for. For example, 665 SNPs could account for 63.52 ± 8.42% of variation in courtship wing movement, 828 accounted for 68.64 ± 6.69% of genital-licking behavior, and 8013 accounted for 78.45 ± 5.97% of courtship approach behavior. The results from both GCTA tests in the DGRP lines support the hypothesis that, at the genetic level, courtship and feeding-related behaviors are associated with more heritable architectures of large effect, even within less-diverged natural populations.

I next used the DGRP lines to query the extent to which genes or genomic loci may affect multiple behavioral traits (pleiotropy) (Greenspan 2004). I exploited the breadth of phenotypic and genomic data available in the DGRP to empirically address this question at three levels: SNPs, genes, and traits. To allow for comparisons of behavior and other trait types, I also conducted a GWAS for 26 morphological traits reported in Vonesch *et al.* (2016) (supplemental methods in File S1 and Table S5). SNPs found to be associated with morphology and behavior at *P* < 1 × 10^−5^ were distributed across the *D. melanogaster* genome, 80 of which were associated with both behavioral and morphological traits (Figure 3C).

With this list of variants, I queried which individual SNPs were associated with multiple behavioral categories. I identified 169 SNPs associated with at least two behavioral measures. These variants largely overlapped within behavioral categories rather than between categories, suggesting that, at the level of individual SNPs, these traits may have largely independent genetic architectures among the DGRP lines (Figure 3D). Many of these SNPs fell within the same genomic regions. Seventy-two genes had at least two SNPs associated with multiple traits, several of which contained a multitude of variants (Figure S2A in File S1).

I then assessed the extent to which behaviorally associated variants may act pleiotropically at the trait level, using the list of 25,919 variants associated with behavior. With this, I correlated the effect sizes of trait-associated SNPs with the effect sizes of those same variants across all other traits. The results of this analysis are summarized in the clustered heatmap in Figure S3 in File S1. In general, I found extensive correlations between behavioral traits, suggesting widespread pleiotropic genetic effects. I also observed several large clusters of highly correlated traits, suggesting a higher-level structure for phenotypic variation based on trait interactions (labeled 1–4 in Figure S3 in File S1). Analyses of genetic correlations using whole-genome SNP information via bivariate GREML paralleled this observation of structured trait correlations (Figure S4 in File S1). The existence of these apparent clusters suggests that, while behavioral categories in the DGRP overlap little in genomic architecture at the individual variant level, there may be common molecular pathways through which different behavioral traits are altered in a correlated fashion.

Finally, I explored pairs of traits with putative directional relationships given the effect sizes of their associated variants. I avoid calling these relationships causal since, given the existence of extensive epistasis and genetic linkage in the DGRP lines, it is difficult to identify individual variants of likely causal effect (Huang *et al.* 2012). I instead sought to elucidate aspects of a directional relationship by discriminating between cases in which a genotype effects multiple traits through different mechanisms *vs.* scenarios where a genotype exerts an effect on a trait through a second, intermediate trait (summarized as *P_1_←G→P_2_* compared to *G→P_1_→P_2_*) (Pickrell *et al.* 2016). In addition to the 87 identified behavioral traits, I included the 26 morphological measures to gather insights into potentially directional relationships between behavior and morphology in the DGRP.

**Figure 4:**
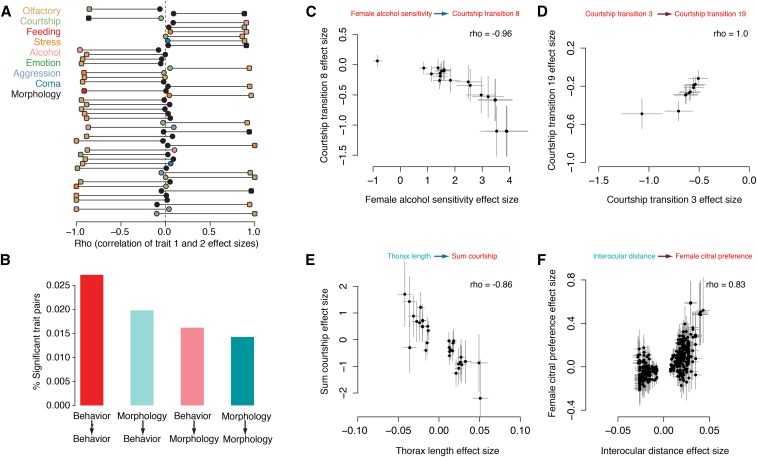
Directional relationships between trait pairs in the *Drosophila* Genetic Resource Panel. (A) Directional trait pairs identified as significant by permutation testing. Plotted are traits where the significant correlation possesses a ρ > 0.85. The significant correlation is represented by a rounded rectangle. (B) Barplot summarizing the number of significant trait pairs identified where the focal trait is either behavioral or morphological, with a correlation with one of these two domains. Behavioral focal traits are colored red and morphological traits are colored blue. (C–F) Scatterplots of the effect sizes for the focal SNPs of example significant trait pairs. SE are plotted as gray lines.

I conducted pairwise tests of each trait at which GWAS variants at the *P* < 5 × 10^−6^ level were identified. Using a permutation-based test, I found 143 trait pairs that showed directionality wherein the correlation of effect sizes was strong and significant in one comparison but not the other (Figure 4A and supplemental methods in File S1).

Trait pairs identified as significant showed an uneven distribution of potential directional effect between behavior and morphology, with the largest amount occurring between pairs of behavioral traits (Figure 4B). Figure 4, C–F highlight examples of these SNP effect size correlations for different behavioral and morphological measures. A particularly interesting connection was found between SNPs associated with EGFR signaling affecting thorax length and the total amount of courtship attempted by male flies (ρ = −0.86, *P* = 8.6 × 10^−8^; supplemental methods in File S1).

The connection between male courtship behaviors and body size has long been recognized in laboratory strains of *Drosophila*, though with little evidence of a molecular basis for this effect (Ewing 1961). In general, I find extensive evidence of both directional (*G→P_1_→P_2_*) and general (*P_1_←G→P_2_*) pleiotropic effects between traits in the DGRP, supporting the notion that the early stages of behavioral diversification involve the role of genes that can affect multiple types of traits. Furthermore, I observe that while variation in behavior across trait categories is associated with nonoverlapping variants, these may occur in common genes and molecular pathways with pleiotropic effects, reflecting suggestions of the existence of phenotypic “hotspots” that are recurrently used by evolution to sculpt phenotypes (Stern and Orgogozo 2008).

Taken together, these results suggest that behavioral traits may respond to evolutionary processes with greater variation than previously appreciated. For example, researchers may now anticipate that assaying a courtship ritual will likely yield a higher genetic effect than, say, variation in a personality trait. These insights are supported by observations that behavioral categories vary in their heritability and genomic architecture during even the earliest stages of diversification within populations. Furthermore, such behaviors are associated with a small number of highly pleiotropic genes and these traits interact, indicating that there are identifiable molecular and phenotypic patterns that govern behavior.

These findings suggest several important caveats and prospects for future behavior genetic studies. First, QTL mapping methods possess inherent limitations in detecting the complete genetic architecture of certain traits. For example, QTL studies are often insensitive to the detection of loci with opposing effects on the trait of interest, thus potentially masking important genetic effects from the researcher’s analysis (Mackay *et al.* 2009). Future studies of the genetic architecture of behavior will thus benefit from integrating QTL methods with results from genome-wide sequencing and genetic interrogations directed by genome editing. Second, a more complete survey of behavioral categories within and across a variety of taxa is needed to confidently establish whether or not the patterns observed in this study are general principles of how behavior evolves. This is compounded by the fact that it is notoriously difficult to overcome issues like the “file drawer problem” in the QTL mapping literature, wherein insignificant results are often under- or nonreported (Rosenthal 1979). While this study took efforts to control for as many aspects of biases in study design and sensitivity as possible, it remains difficult to fully control for publication biases in the meta-analysis of QTL experiments. Finally, empirical tests in the field and laboratory may offer a deeper understanding of the extent to which courtship and feeding behaviors respond uniquely to selective pressures, and which evolutionary and ecological mechanisms may account for this phenomenon. Expanding on this with the tools and data now becoming available, behavioral biology may begin to produce a more nuanced and predictive understanding of the interplay of genetic forces governing the evolution of behavior.

## Supplementary Material

Supplemental material is available: online at www.genetics.org/lookup/suppl:/doi:10.1534/genetics.118.300712/-/DC1.
