# Supplementary material for: Assessing the Genetic Landscape of Animal Behavior
Source: Genetics. 2018 Mar 21;209(1):223–32. doi: 10.1534/genetics.118.300712 (PMC5937184; doi:10.1534/genetics.118.300712)
Supplement: Supplementary file 1 [file 223FileS1.pdf]

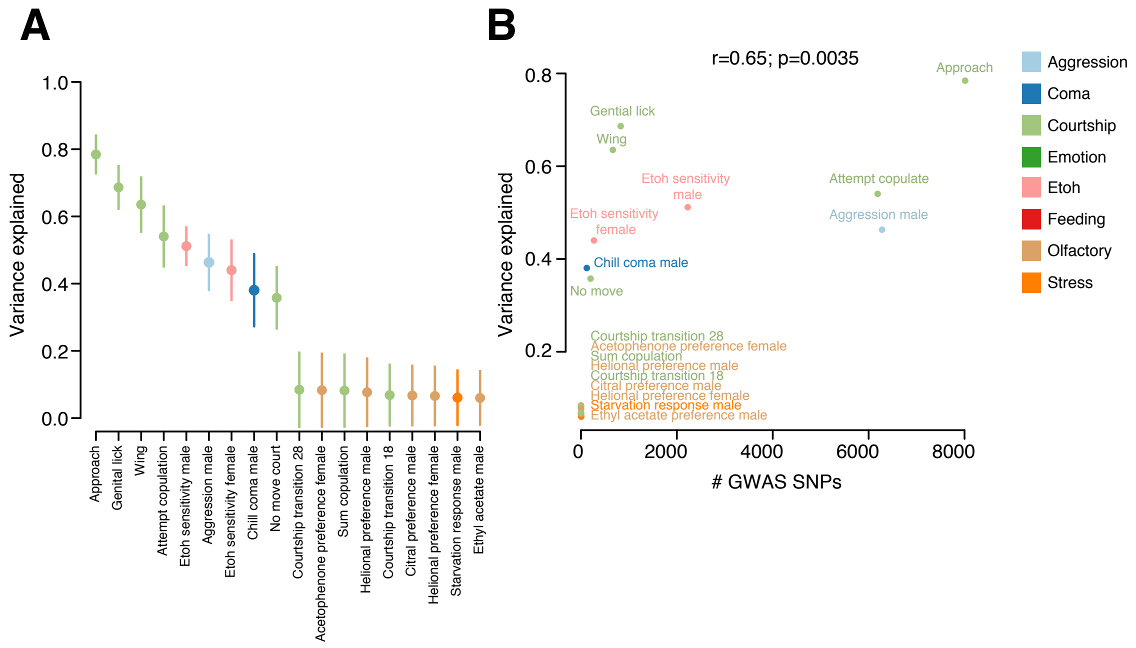

Figure S1 | GCTA analyses using trait-specific SNP sets.

(A) Genomic heritability estimates (Vg/Vp) from GCTA using only GWAS significant SNPs for each trait. Plotted are the 16 measures identified as significant (p-value < 0.05), colored by behavioral category. (B) Scatterplot of these same traits comparing the number of GWAS significant SNPs used for GCTA to the amount of variance explained. A Pearson correlation was used to calculate the association.

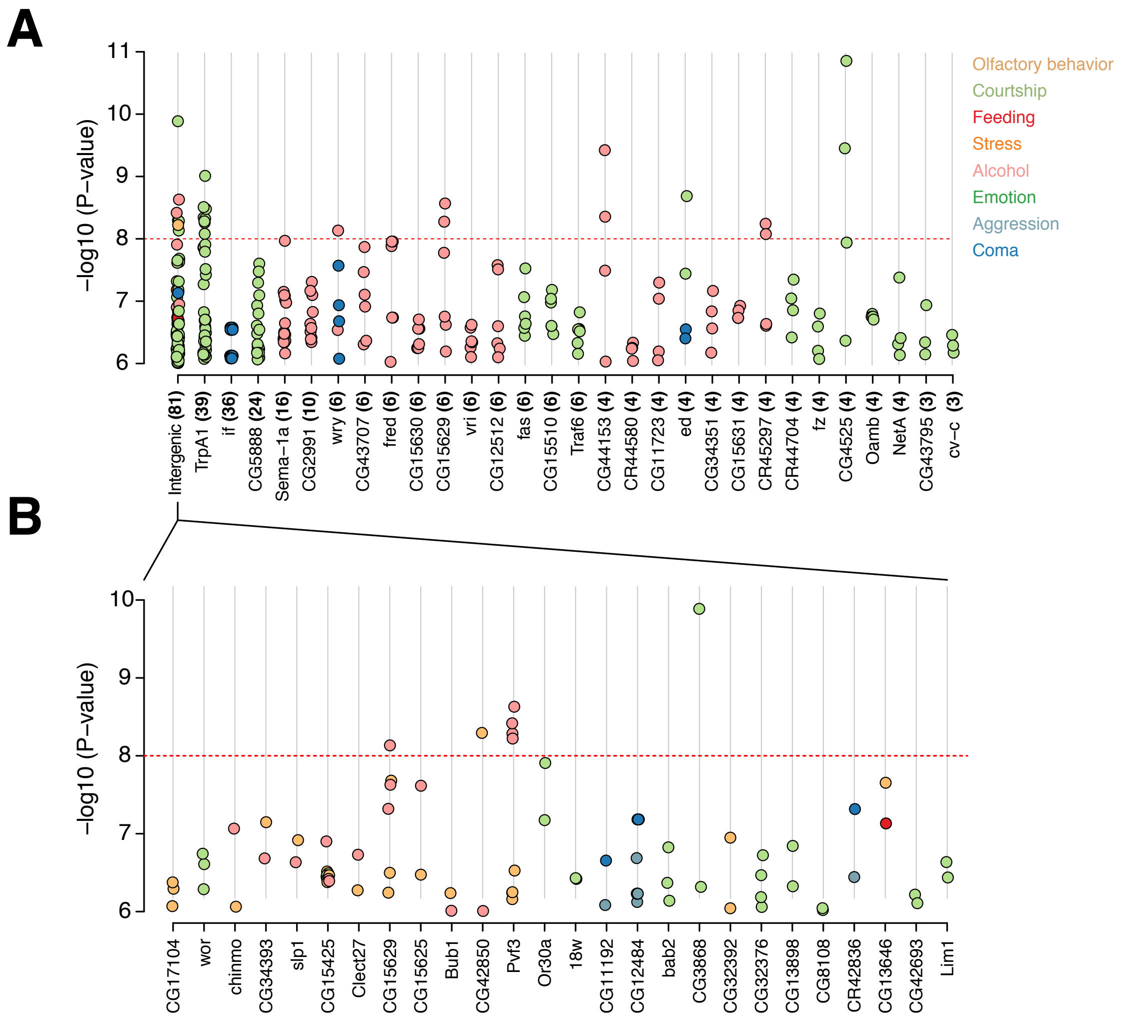

Figure S2 | Genes associated with multiple traits.

(A) Stripchart of genes containing variants associated with at least two behavioral measures. Each category is color-coded. Genome-wide significance ($p < 5 \times 10^{-8}$) is marked with the red dashed line. (B) Stripchart of the genes nearest each of the 81 intergenic variants identified to be associated with at least two behavioral measures. Note that this is a subset of Fig. S6A.

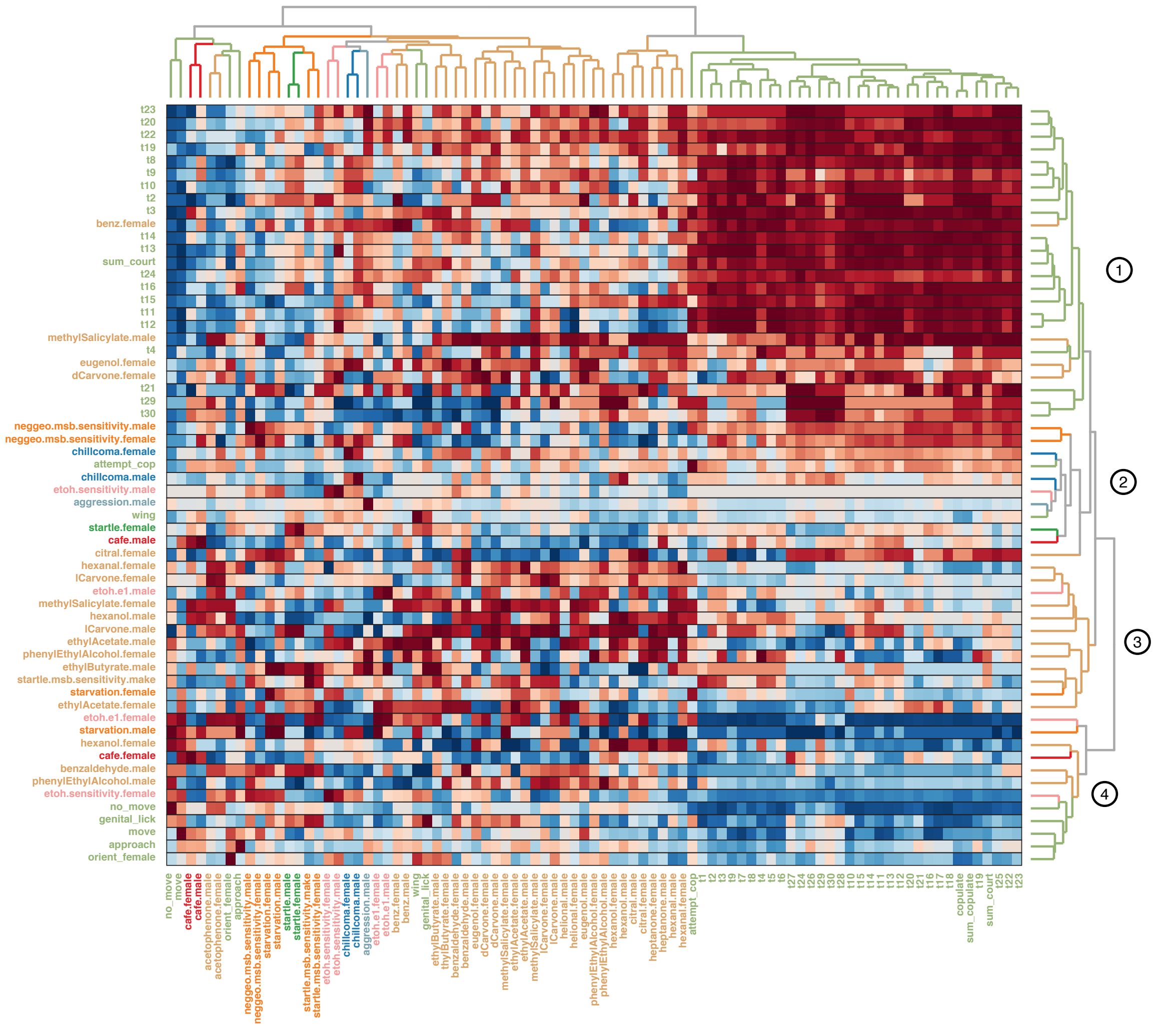

Figure S3 | Heatmap of effect size correlations for GWAS significant SNPs.

Behavioral measures are labeled following the original conventions reported by the authors in refs. *13-21*. The traits are color coded by category, following the scheme in Fig. S4. Red represents a positive rho (positive correlation), blue represents a negative rho (correlation), and darkness encodes strength of correlation.

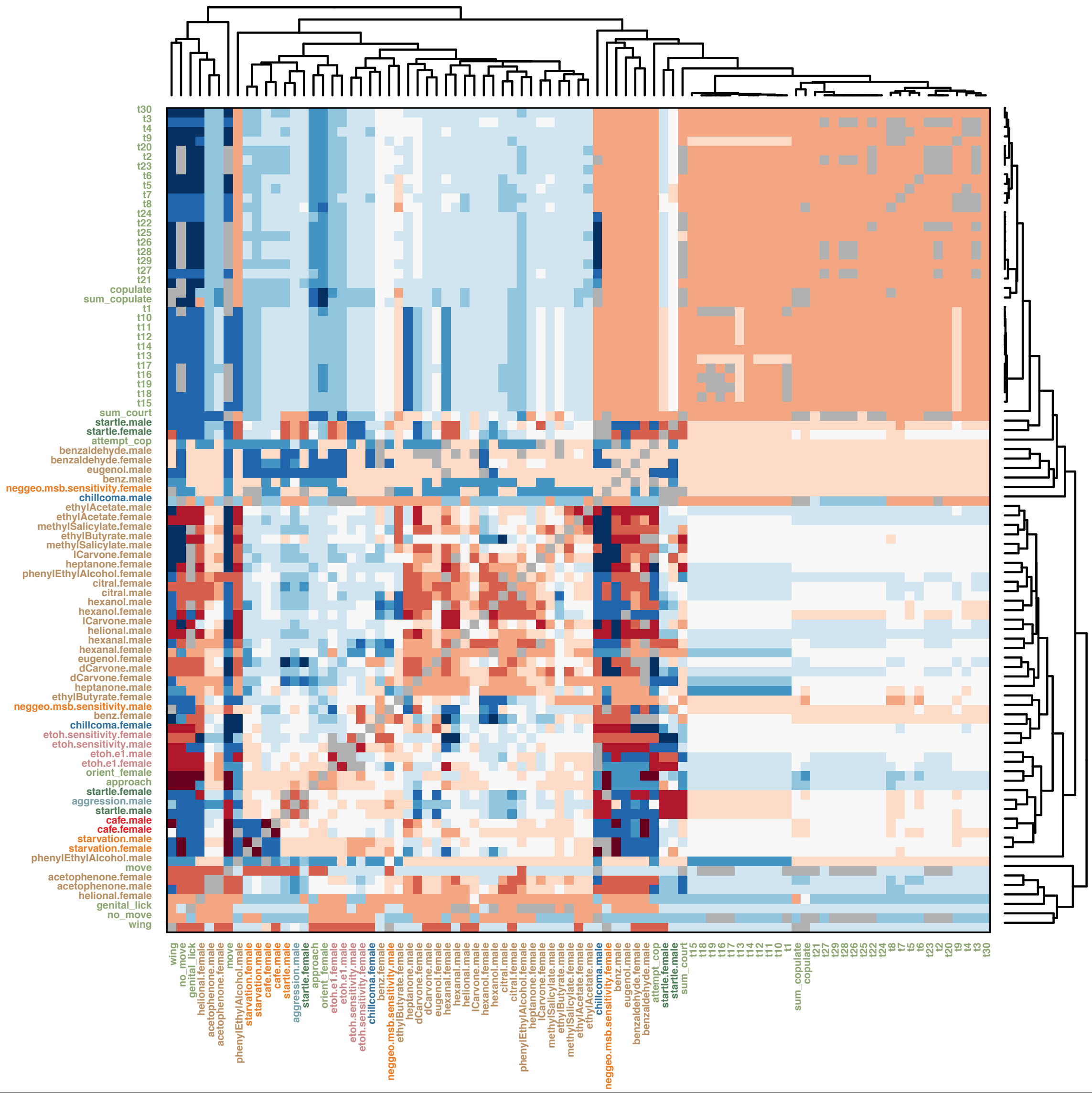

Figure S4 | Heatmap of genetic correlations between DGRP traits.

Heatmap colors represent genetic correlations calculated using bivariate GREML. Red represents a positive correlation; blue represents a negative correlation; grey represents NA. Traits were hierarchically clustered before plotting. The traits are color coded by category, following the scheme in Fig. S4

Rank	Sample Size	Generations	Inter-/intraspecific	DIC
1				4462.879
2				4462.908
3				4637.916
4				4637.925
5				4655.640
6				4723.008
7				5191.479

Table S1 | MCMCglmm models compared.

Represented in the table are the ranks of the 7 models tested for MCMCglmm analysis of QTL effect sizes. In each model behavioral category was used a fixed effect (not shown) while sample size, generation time, and inter-/intraspecific crossing scheme were used as random effects. Grey boxes underneath the three possible random effects represent inclusion in the given model represented by that row. The models are ranked by their deviance information criterion (DIC) in the last column.

Fixed effect	Estimate	Standard error	t-value
Circadian	-4.070	4.517	-0.901
Courtship	20.131	5.984	3.364
Emotion	6.490	4.211	1.541
Feeding	13.721	4.494	3.053
Learning	2.579	3.010	0.857
Motor	5.039	2.644	1.906
Parental	-1.451	2.211	-0.656
Social	-2.525	3.316	-0.762
Temperament	3.477	3.285	1.058

Table S2 | Results of linear mixed effect model from lme4.

Presented are results from a linear mixed effect model using behavioral category as a fixed effect and generation time and sample size as random effects (same as best ranked model in the MCMCglmm analyses presented in Table S1 and Figure 2).

Trait	Observed mean	#of observations	*p*-value
Circadian	3.369	40	1.000
Courtship	20.932	115	<0.0005
Emotion	4.767	229	1.000
Feeding	19.297	32	<0.0005
Learning	6.69	20	0.842
Motor	10.729	76	0.061
Parental	5.599	17	0.938
Social	1.544	58	1.000
Temperament	10	8	0.342

Table S3 | Permutation tests of behavioral trait effect sizes.

For each behavioral trait the observed mean of reported effect sizes and number of observations of that trait in the data set are presented. *P*-values were calculated by comparing the observed means to a null distribution produced from 10,000 permutations of the effect size data.

Sources and rationale for estimation of evolutionary divergence

Zebrafish

According to Whitelely et al. (2011) separation between AB lab strain (clusters with Indian populations phylogenetically) and Bengladesh clades occurred 2-3 mya. Estimated divergence at 2 mya between AB and Bengladesh populations used for the Wright 2006 study. Average generation time estimate of 3 months was used, yielding an evolutionary divergence of 8,000,000 generations.

White fish

Rogers et al. (2007) sampled “dwarf” and “normal” ecotypes of whitefish from two lakes in which these ecotypes have been under postglacial isolation since the Pleistocene. Given the suggested age of these lakes and the ecotypes contained within as laid out in Rogers et al. (2007) and Lu and Bernatchez (1999) an estimated divergence time of 18,000 years was used. Since the “dwarf” ecotype reaches maturity by 1 year whereas the “normal” ecotype reaches maturity at 2 years a conservative estimated generation time of 2 years was used, yielding an evolutionary divergence of 9,000 generations.

Three-spined stickleback

Greenwood et al (2013; 2015) crossed marine three-spined sticklebacks from Japan with freshwater populations from North America. Freshwater populations have been formed through many migrations from marine to freshwater ecosystems since the last glacial retreat ~12,000 years ago (Greenwood et al 2013). Since it is difficult to determine the relationship between Japanese populations and the ancestral marine species that first inhabited the lakes sampled for this study

the estimated divergence time of 12,000 years was used, though this number may turn out to be too recent of a date ultimately. A reported generation time of 1 year was used (Shapiro et al 2006).

Solenopsis invicta

An exact date of the divergence time between SB and Sb individuals is currently unavailable. Wang et al. 2013 do propose that recombination suppression of the social chromosome appears to have occurred ~390,000 years ago. Using this date as a rough estimate of the divergence between the two social organizations, with an estimated generation time of around 6 years taken from measures of colony age (Tschinkel 1987), yields a total of 6,500 generations.

Sheep (Romane lambs)

Hazard et al. 2014 used half-sib Romane lambs which are a fixed crossbreed between Romanov x Berrichon du Cher. Since the crosses tested were between siblings evolutionary divergence was measured as 1 generation. A generation time of 6 years was used.

Rat (Albert et al. 2009)

Albert et al. 2009 used rat lines selected for tameness for 64 generations beginning in 1972, yielding an evolutionary divergence time of 37 years (estimated from the publication date of 2009).

Rat (Alcohol-preferring (P) x alcohol-nonpreferring (NP); e.g. Bice/Carr 1998)

According to Bice et al. 1998 rats were selected for alcohol preferring and non-preferring traits for 30 generations, followed by 19 generations of inbreeding, for a total of 49 generations with an estimated time of 25 years (given 6 month generation time).

Rat (Roman high x low avoidance strains; e.g. Fernandez-Teruel et al 2002)

RLA and RHA rats were selected for high and low avoidance over 5 generations (Bignami 1965) and then inbred beginning in 1993. Estimated number of years to be 40 since start of breeding until year of study, with a total number of generations estimated at 80 (6 month generation time).

Rat (WKY x WKHA strains; e.g. Moisan 1996)

WKHA strain was established in 1980 from a cross of WKY and SHR strains (Drolet et al. 2002). The estimated divergence time was set at 16 years with an estimated generation number of 32.

Rat (LEW x SHR strains; e.g. Ramos 1999)

Currently not able to resolve relationship between LEW and SHR

Pig (Meishan x Large white breeds/Meishan x Pietrain breeds; e.g. Desautes 2002 and Reiner et al. 2009)

Desautes et al (2002) crossed Meishan and Large White breed pigs. Meishan is a Chinese breed while Large White is European, therefore suggesting that these breeds are separated by the known domestication split between Eastern and Western breeds at ~10,000 years ago (Li et al. 2014). The Meishan and Pietrain cross used in Reiner et al. 2009 represents the same evolutionary distance. Generation time of pigs was estimated to be 1 year.

Ruff (Kupper et al. 2014)

Like the SB/Sb social chromosome in fire ants dating the divergence time between ruff mating morphs exactly is difficult. The authors predict the date of the inversion occurred ~3.8 MYA. Following Verkuil et al. 2014 a generation time of 3 years was used.

Peromyscus (maniculatus x polionotus)

Using mtDNA data Avise et al. 1983 provisionally placed the split between Peromyscus maniculatus and Peromyscus polionotus at 1.5 million years ago. This is a rough estimate and will be benefitted by future work incorporating the larger molecular data sets now available. Generation time was estimated at 3 months according to Joyner et al. 1998.

Pea aphid (red clover x alfalfa biotypes)

Cailalaud and Via (2012) mapped QTL affecting the feeding behavior of red clover x alfalfa biotypes. Though an exact divergence time for these two biotypes is not known molecular phylogenetic estimates place the divergence of pea aphid biotypes after the Pleistocene concurrent with the advent of agriculture. A relatively conservative estimated of a divergence time of 7,000 years was used for the divergence between the two biotypes as motivated by Peccoud et al's (2009) speculation. Since pea aphids are believed to be capable of producing 12-15 generations per year (http://ag.arizona.edu/pubs/insects/az1044/) an estimated generation 1.125 months was used.

Nine-spined stickleback (marine x freshwater)

Laine et al. (2014) crossed a marine population from the Baltic Sea with a pond population from Northeastern Finland. Similar to three-spined sticklebacks in North America the Northern

European freshwater populations of nine-spined sticklebacks likely diverged from marine species beginning around 12,000 years ago (Shikano et al 2010). A generation time of 1 year was used.

Nasonia wasps (*oneida* x *giraulti*)

Werren et al. 2010 estimate the divergence time between *Nasonia Oneida* and *Nasonia giraulti* at 410,000 years ago. Werren and Loehlin 2009 report a generation time of 14 days for *Nasonia* species.

Mice

All divergence dates, unless otherwise noted, for laboratory mouse strains were obtained from the genealogy available from the Mouse Genome Informatics website (http://www.informatics.jax.org/mgihome/genealogy/). This genealogy was originally produced by Beck et al. 2000.

Mice (C57-related strains x Castle's mice; e.g. C57BL6xA/J in Bruining et al 2015)

According to Beck et al's (2000) genealogy C57-related strains and Castle's mice split when Lathrop sent Castle mice between 1903 and 1915. A/J was derived from the original DBA stock in 1909, placing the most recent common ancestor at 1909, yielding a divergence time of 106 years. A generation time of 3 months is assumed for all mouse strain crosses.

Mice (C57BL6 x C57BLJ)

Most recent common ancestor of 1921.

Mice (C57BL6 x DBA2J)

Most recent common ancestor of 1909.

Mice (C57BL6 x BALB/cJ)

Most recent common ancestor of 1909.

Mice (NZB/BINJ x A/J)

Most recent common ancestor of 1909.

Mice (C3H/HeJ x C57BL6)

Most recent common ancestor of 1909.

Mice (A/J x CBA/J)

Most recent common ancestor of 1909.

Heterogeneous stock mice (HS)

HS mice are derived from crosses of eight inbred strains: A/J, AKR/J, BALB/cJ, C3H/HeJ, C57BL/6J, CBA/J, DBA/2J and LP/J. HS mice I originally created in the 80s and has been maintained for over 50 generations. The most recent common ancestor of the original inbred strains occurred in 1909.

Mice (ILS ISS)

McClearn and Kakihana (1973) bred the long-sleep and short-sleep lines for use in alcohol addiction research. Most recent common ancestor estimated at 1973.

Mice (BTBR x C57BL/6J)

Most recent common ancestor of 1909.

Mice (C57BL/6 x NZB/BINJ)

Most recent common ancestor of 1909.

Mice (C57L/J x C3H/HeJ)

Most recent common ancestor of 1909.

Mice (SM/J x LG/J)

Most recent common ancestor of 1931.

Mice (A/J x BALB/cJ)

Most recent common ancestor of 1921.

Mice (SM/J x A/J)

Most recent common ancestor of 1909.

Mice (DeFries high x DeFries low)

Most recent common ancestor of 1964.

Mice (C57BL/6 x C3H/He)

Most recent common ancestor of 1909.

Wax moths (Florida *Achroia grisella* x Kansas *Achroia grisella*)

Limousin et al (2012) crossed wax moths raised in Florida and Kansas to map signaling traits. Wax moths were first seen in North America in 1806 and presumably the divergence between Florid and Kansas populations has occurred since then. I estimated a divergence time of 200 years for these populations and used a generation time of 4 months.

Wax moths (Louisiana *Achroia grisella* x French *Achroia grisella*)

Alem et al (2013) crossed wax moths from Louisiana with a population from France. Given the origin of North American wax moths at somewhere likely shortly before 1806 I used an estimated divergence between these two populations of the year 1700 to account for possible population divergence of European populations before migration to North America. This number is currently very speculative-the real date could be either much older or more recent-and will need to molecular phylogenomic analyses of worldwide *Achroia grisella* populations for resolution.

Medaka (HNI-II x Hd-rR-II1)

HNI-II medaka strain was derived from the Northern population while Hd-rR_II1 was derived from the Southern medaka population. The Northern and Southern populations diverged 4 millions years ago (Kirchmaier et al. 2015). Medaka have a generation time of 8-12 weeks depending on strain.

Japanese quail (High x low tonic immobility strains)

Japanese quail selected for high or low tonic immobility were selected for QTL mapping by Recoquillay et al (2015) after the 49th generation of selection. A generation time of 4 months was used (Minvielle & Oguz 2002).

Horse (Brard and Ricard 2015)

Due to a pooling of samples from different breeds used for this GWAS it is difficult to establish an estimated divergence time at the current moment.

Fugu (*Takifugu rubripes* x *Takifugu niphobles*)

Takifugu rubripes and *niphobles* diverged around 4.7 mya (Yamanoue et al. 2008). A generation time of 2 years was used (Kai et al. 2011).

Drosophila (*Drosophila simulans* x *sechellia*)

Garrigan et al (2012) estimated the date of divergence for the *simulans* clade at ~242,000 years ago. I used an estimated generation time of 3 weeks.

Drosophila (*Drosophila virilis* (England) x *Drosophila virilis* (Japan))

Huttunen et al (2003) crossed *virilis* species from England and Japan. Morales-Hojas et al. 2011 calculate a likely migration into Eurasia for the *virils* phylad as occurring around 2 MYA while most spreads westward likely occurred with the advent of domestication in the post-glacial period

(Mirol et al. 2008). I used a measure in the middle of these two extremes, 500,000 years, for the divergence of the populations. I used an estimated generation time of 3 weeks.

Drosophila (*Drosophila sanotmea* x *yakuba*)

Santomea and *yakuba* diverged around 400,000 years ago (Cande et al 2012).

Drosophila (*Drosophila melanogaster Oregon-R* x *melanogaster 2b*)

Oregon-R is a North American strain while 2b is from Russia. I date the divergence of the two strains at around 10,000 years ago following Arguello et al. 2016, coinciding with the spread of human civilization.

Drosophila (*Drosophila elegans* x *gunungcola*)

Drosophila elegans and *gunungcola* diverged 2-2.8 mya (Prud'homme et al. 2006).

Dogs

Ha et al. (2015) conducted a GWAS across dog breeds, the most recent common ancestor of which is estimated between 9,000 and 34,000 years ago. I used an intermediate estimate of 15,000 years and a generation time of 2 years.

Laupala crickets (*Laupala paranigra* x *Laupala kohalensis*)

Mendelson and Shaw (2005) estimate the divergence between *kohalensis* and *paranigra* to be 0.43 mya. I used a generation time of 3 months.

Chicken (Red junglefowl x white leghorn)

Schutz et al (2002) crossed the likely ancestral chicken species, red junglefowl, with a domestic breed. Chicken domestication began 5,400 years before present (Storey et al. 2012). I used a generation time of 6 months.

Cave fish

Surface and cave populations of Mexican cave fish are estimated to begin divergence between 10,000 and several million years ago (Protas 2012). I used an estimate of 100,000 years ago and a generation time of 6 months (Jeffery 2001).

***C. elegans* (N2 x CB4856)**

Cutter 2008 estimates that *C. elegans* and its sister species *C. briggsae* diverged ~14 MYA with a generation time of 60-days.

***Apis mellifera* (Fast x slow learners i.e. Chandra et al. 2001)**

Chandra et al. (2001) used strains of honeybees bred for learning ability over 2 generations. A generation time of 1 year was used.

***Apis mellifera* (High-pollen x low-pollen i.e. Hunt et al. 1995)**

Hunt et al. (1995) bred strains of honeybees selecting for high-pollen and low-pollen hoarding for two generations before conducting a backcross for mapping. Rueppel et al. (2004) used the same strains after 18 generations of breeding.

***Apis mellifera* (African drone x European queen i.e. Hunt et al. 1998, 1999)**

Hunt et al. (1998) crossed an *A. mellifera* queen with a drone from the African subspecies *A. mellifera scutellata*. These two populations presumably diverged ~300,000 years ago (Wallberg et al. 2014).

***Apis mellifera* (High *Varroa* expression x low *Varroa* expression)**

Tsuruda et al (2012) crossed colonies with high or low expression of *Varroa* sensitive behaviors and mapped a backcross of F1.

Japanese three-spined stickleback (Japan Sea x Pacific)

Kitano et al. (2009) crossed three-spined stickleback from the Japan Sea with Pacific individuals. They report a divergence time of 1.5-2 MYA. A generation time of 1 year was used.

Supplementary references:

1. Albert FW, Carlborg O, Plyusnina I, Besnier F, Hedwig D, Lautenschlager S, et al. Genetic Architecture of Tameness in a Rat Model of Animal Domestication. Genetics. 2009;182(2):541-54. doi: 10.1534/genetics.109.102186. PubMed PMID: WOS:000270213900013.
2. Alem S, Streiff R, Courtois B, Zenboudji S, Limousin D, Greenfield MD. Genetic architecture of sensory exploitation: QTL mapping of female and male receiver traits in an acoustic moth. J Evolution Biol. 2013;26(12):2581-96. doi: 10.1111/jeb.12252. PubMed PMID: WOS:000327115900005.
3. Alexander RC, Wright R, Freed W. Quantitative trait loci contributing to phencyclidine-induced and amphetamine-induced locomotor behavior in inbred mice. Neuropsychopharmacology. 1996;15(5):484-90. doi: Doi 10.1016/S0893-133x(96)00058-9. PubMed PMID: WOS:A1996VP99300007.
4. Arguello JR, Cardoso-Moreira M, Grenier JK, Gottipati S, Clark AG, Benton R. Extensive local adaptation within the chemosensory system following Drosophila melanogaster's global expansion. Nat Commun. 2016;7:ncomms11855. doi: 10.1038/ncomms11855. PubMed PMID: 27292132; PubMed Central PMCID: PMCPMC4910016.
5. Arya GH, Magwire MM, Huang W, Serrano-Negron YL, Mackay TF, Anholt RR. The genetic basis for variation in olfactory behavior in Drosophila melanogaster. Chem Senses. 2015;40(4):233-43. doi: 10.1093/chemse/bjv001. PubMed PMID: 25687947; PubMed Central PMCID: PMCPMC4398050.
6. Avise JC, Shapira JF, Daniel SW, Aquadro CF, Lansman RA. Mitochondrial DNA

differentiation during the speciation process in Peromyscus. Mol Biol Evol. 1983;1(1):38-56. PubMed PMID: 6400647.

7. Beck JA, Lloyd S, Hafezparast M, Lennon-Pierce M, Eppig JT, Festing MF, et al. Genealogies of mouse inbred strains. Nat Genet. 2000;24(1):23-5. doi: 10.1038/71641. PubMed PMID: 10615122.
8. Belknap JK, Richards SP, OToole LA, Helms ML. Short-term selective breeding as a tool for QTL mapping: Ethanol preference drinking in mice. Behavior Genetics. 1997;27(1):55-66. doi: Doi 10.1023/A:1025615409383. PubMed PMID: WOS:A1997WX87700006.
9. Bendesky A, Pitts J, Rockman MV, Chen WC, Tan MW, Kruglyak L, et al. Long-Range Regulatory Polymorphisms Affecting a GABA Receptor Constitute a Quantitative Trait Locus (QTL) for Social Behavior in Caenorhabditis elegans. Plos Genetics. 2012;8(12). doi: ARTN e100315710.1371/journal.pgen.1003157. PubMed PMID: WOS:000312905600053.
10. Bendesky A, Tsunozaki M, Rockman MV, Kruglyak L, Bargmann CI. Catecholamine receptor polymorphisms affect decision-making in C. elegans. Nature. 2011;472(7343):313-U207. doi: 10.1038/nature09821. PubMed PMID: WOS:000289724600032.
11. Bergeson SE, Helms ML, O'Toole LA, Jarvis MW, Hain HS, Mogil JS, et al. Quantitative trait loci influencing morphine antinociception in four mapping populations. Mammalian Genome. 2001;12(7):546-53. doi: DOI 10.1007/s003350020022. PubMed PMID: WOS:000169627100010.
12. Berrettini WH, Ferraro TN, Alexander RC, Buchberg AM, Vogel WH. Quantitative

Trait Loci Mapping of 3 Loci Controlling Morphine Preference Using Inbred Mouse Strains. Nature Genetics. 1994;7(1):54-8. doi: DOI 10.1038/ng0594-54. PubMed PMID: WOS:A1994NJ84200013.

13. Bice P, Foroud T, Bo RH, Castelluccio P, Lumeng L, Li TK, et al. Genomic screen for QTLs underlying alcohol consumption in the P and NP rat lines. Mammalian Genome. 1998;9(12):949-55. doi: DOI 10.1007/s003359900905. PubMed PMID: WOS:000077934900005.
14. Bignami G. Selection for high rates and low rates of avoidance conditioning in the rat. Anim Behav. 1965;13(2):221-7. PubMed PMID: 5835838.
15. Blizard DA, Kotlus B, Frank ME. Quantitative trait loci associated with short-term intake of sucrose, saccharin and quinine solutions in laboratory mice. Chemical Senses. 1999;24(4):373-85. doi: DOI 10.1093/chemse/24.4.373. PubMed PMID: WOS:000082123400002.
16. Brard S, Ricard A. Genome-wide association study for jumping performances in French sport horses. Animal Genetics. 2015;46(1):78-81. doi: 10.1111/age.12245. PubMed PMID: WOS:000348677900013.
17. Brodkin ES, Goforth SA, Keene AH, Fossella JA, Silver LM. Identification of quantitative trait loci that affect aggressive behavior in mice. Journal of Neuroscience. 2002;22(3):1165-70. PubMed PMID: WOS:000173660800065.
18. Bruining H, Matsui A, Oguro-Ando A, Kahn RS, van't Spijker HM, Akkermans G, et al. Genetic Mapping in Mice Reveals the Involvement of Pcdh9 in Long-Term Social and Object Recognition and Sensorimotor Development. Biol Psychiat. 2015;78(7):485-95. doi: 10.1016/j.biopsych.2015.01.017. PubMed PMID:

WOS:000360974400008.

19. Bryant CD, Kole LA, Guido MA, Sokoloff G, Palmer AA. Congenic dissection of a major QTL for methamphetamine sensitivity implicates epistasis. Genes Brain Behav. 2012;11(5):623-32. doi: 10.1111/j.1601-183X.2012.00795.x. PubMed PMID: WOS:000305905700014.

20. Buck K, Lischka T, Dorow J, Crabbe J. Mapping quantitative trait loci that regulate sensitivity and tolerance to quinpirole, a dopamine mimetic selective for D-2/D-3 receptors. American Journal of Medical Genetics. 2000;96(5):696-705. doi: Doi 10.1002/1096-8628(20001009)96:5<696::Aid-Ajmg17>3.0.Co;2-6. PubMed PMID: WOS:000089677400017.

21. Buck KJ, Metten P, Belknap JK, Crabbe JC. Quantitative trait loci involved in genetic predisposition to acute alcohol withdrawal in mice. Journal of Neuroscience. 1997;17(10):3946-55. PubMed PMID: WOS:A1997WX66100049.

22. Cabib S, Bonaventura N. Parallel strain-dependent susceptibility to environmentally-induced stereotypies and stress-induced behavioral sensitization in mice. Physiology & Behavior. 1997;61(4):499-506. doi: Doi 10.1016/S0031-9384(96)00463-5. PubMed PMID: WOS:A1997WT05500004.

23. Caillaud MC, Via S. Quantitative genetics of feeding behavior in two ecological races of the pea aphid, Acyrthosiphon pisum. Heredity. 2012;108(3):211-8. doi: 10.1038/hdy.2011.24. PubMed PMID: WOS:000300596100007.

24. Caldarone B, Saavedra C, Tartaglia K, Wehner JM, Dudek BC, Flaherty L. Quantitative trait loci analysis affecting contextual conditioning in mice. Nature Genetics. 1997;17(3):335-7. doi: DOI 10.1038/ng1197-335. PubMed PMID:

WOS:A1997YD66700031.

25. Cande J, Andolfatto P, Prud'homme B, Stern DL, Gompel N. Evolution of multiple additive loci caused divergence between Drosophila yakuba and D. santomea in wing rowing during male courtship. Plos One. 2012;7(8):e43888. doi: 10.1371/journal.pone.0043888. PubMed PMID: 22952802; PubMed Central PMCID: PMCPMC3431401.

26. Carr LG, Foroud T, Bice P, Gobbett T, Ivashina J, Edenberg H, et al. A quantitative trait locus for alcohol consumption in selectively bred rat lines. Alcoholism-Clinical and Experimental Research. 1998;22(4):884-7. doi: Doi 10.1097/00000374-199806000-00017. PubMed PMID: WOS:000074294100019.

27. Chandra SB, Hunt GJ, Cobey S, Smith BH. Quantitative trait loci associated with reversal learning and latent inhibition in honeybees (Apis mellifera). Behav Genet. 2001;31(3):275-85. PubMed PMID: 11699600.

28. Chang CC, Chow CC, Tellier LC, Vattikuti S, Purcell SM, Lee JJ. Second-generation PLINK: rising to the challenge of larger and richer datasets. Gigascience. 2015;4:7. doi: 10.1186/s13742-015-0047-8. PubMed PMID: 25722852; PubMed Central PMCID: PMCPMC4342193.

29. Cohen RM, Kang A, Gulick C. Quantitative trait loci affecting the behavior of A/J and CBA/J intercross mice in the elevated plus maze. Mammalian Genome. 2001;12(7):501-7. doi: DOI 10.1007/s00335-001-2047-5. PubMed PMID: WOS:000169627100003.

30. Cunningham CL. Localization of Genes Influencing Ethanol-Induced Conditioned Place Preference and Locomotor-Activity in Bxd Recombinant Inbred Mice.

Psychopharmacology. 1995;120(1):28-41. doi: Doi 10.1007/Bf02246142. PubMed PMID: WOS:A1995RJ23700004.

31. Cutter AD. Divergence times in Caenorhabditis and Drosophila inferred from direct estimates of the neutral mutation rate. Mol Biol Evol. 2008;25(4):778-86. doi: 10.1093/molbev/msn024. PubMed PMID: 18234705.

32. de Mooij-van Malsen JG, van Lith HA, Laarakker MC, Brandys MK, Oppelaar H, Collier DA, et al. Cross-species genetics converge to TLL2 for mouse avoidance behavior and human bipolar disorder. Genes Brain Behav. 2013;12(6):653-7. doi: 10.1111/gbb.12055. PubMed PMID: WOS:000322546900007.

33. Demarest K, Koyner J, McCaughran J, Cipp L, Hitzemann R. Further characterization and high-resolution mapping of quantitative trait loci for ethanol-induced locomotor activity. Behavior Genetics. 2001;31(1):79-91. doi: Doi 10.1023/A:1010261909853. PubMed PMID: WOS:000170584500008.

34. Demarest K, McCaughran J, Mahjubi E, Cipp L, Hitzemann R. Identification of an acute ethanol response quantitative trait locus on mouse chromosome 2. Journal of Neuroscience. 1999;19(2):549-61. PubMed PMID: WOS:000077966400005.

35. Desautes C, Bidanel JP, Milan D, Iannuccelli N, Amigues Y, Bourgeois F, et al. Genetic linkage mapping of quantitative trait loci for behavioral and neuroendocrine stress response traits in pigs. Journal of Animal Science. 2002;80(9):2276-85. PubMed PMID: WOS:000177901100010.

36. Diao WW, Mousset M, Horsburgh GJ, Vermeulen CJ, Johannes F, van de Zande L, et al. Quantitative Trait Locus Analysis of Mating Behavior and Male Sex Pheromones in Nasonia Wasps. G3-Genes Genom Genet. 2016;6(6):1549-62. doi:

10.1534/g3.116.029074. PubMed PMID: WOS:000377821600007.

37. Drolet G, Proulx K, Pearson D, Rochford J, Deschepper CF. Comparisons of behavioral and neurochemical characteristics between WKY, WKHA, and Wistar rat strains. Neuropsychopharmacology. 2002;27(3):400-9. doi: 10.1016/S0893-133X(02)00303-2. PubMed PMID: 12225697.

38. Eisener-Dorman AF, Lawrence DA, Bolivar VJ. Behavioral and genetic investigations of low exploratory behavior in Il18r1(-/-) mice: We can't always blame it on the targeted gene. Brain Behavior and Immunity. 2010;24(7):1116-25. doi: 10.1016/j.bbi.2010.05.002. PubMed PMID: WOS:000282246800012.

39. Erwin VG, Radcliffe RA, Gehle VM, Jones BC. Common quantitative trait loci for alcohol-related behaviors and central nervous system neurotensin measures: Locomotor activation. Journal of Pharmacology and Experimental Therapeutics. 1997;280(2):919-26. PubMed PMID: WOS:A1997WG58000049.

40. Fernandez-Teruel A, Escorihuela RM, Gray JA, Aguilar R, Gil L, Gimenez-Llort L, et al. A quantitative trait locus influencing anxiety in the laboratory rat. Genome Research. 2002;12(4):618-26. doi: 10.1101/gr.203402. PubMed PMID: WOS:000174971100011.

41. Foreman JE, Lionikas A, Lang DH, Gyekis JP, Krishnan M, Sharkey NA, et al. Genetic architecture for hole-board behaviors across substantial time intervals in young, middle-aged and old mice. Genes Brain Behav. 2009;8(7):714-27. doi: 10.1111/j.1601-183X.2009.00516.x. PubMed PMID: WOS:000270434700007.

42. Gaertner BE, Parmenter MD, Rockman MV, Kruglyak L, Phillips PC. More Than the Sum of Its Parts: A Complex Epistatic Network Underlies Natural Variation in

Thermal Preference Behavior in Caenorhabditis elegans. Genetics. 2012;192(4):1533-+. doi: 10.1534/genetics.112.142877. PubMed PMID: WOS:000311960500026.

43. Gaertner BE, Ruedi EA, McCoy LJ, Moore JM, Wolfner MF, Mackay TF. Heritable variation in courtship patterns in Drosophila melanogaster. G3 (Bethesda). 2015;5(4):531-9. doi: 10.1534/g3.114.014811. PubMed PMID: 25650358; PubMed Central PMCID: PMCPMC4390569.

44. Garlapow ME, Huang W, Yarboro MT, Peterson KR, Mackay TF. Quantitative Genetics of Food Intake in Drosophila melanogaster. Plos One. 2015;10(9):e0138129. doi: 10.1371/journal.pone.0138129. PubMed PMID: 26375667; PubMed Central PMCID: PMCPMC4574202.

45. Garrigan D, Kingan SB, Geneva AJ, Andolfatto P, Clark AG, Thornton KR, et al. Genome sequencing reveals complex speciation in the Drosophila simulans clade. Genome Res. 2012;22(8):1499-511. doi: 10.1101/gr.130922.111. PubMed PMID: 22534282; PubMed Central PMCID: PMCPMC3409263.

46. Gershenfeld HK, Neumann PE, Li XH, St Jean PL, Paul SM. Mapping quantitative trait loci for seizure response to a GABA(A) receptor inverse agonist in mice. Journal of Neuroscience. 1999;19(10):3731-8. PubMed PMID: WOS:000080162400010.

47. Gershenfeld HK, Paul SM. Mapping quantitative trait loci for fear-like behaviors in mice. Genomics. 1997;46(1):1-8. doi: DOI 10.1006/geno.1997.5002. PubMed PMID: WOS:A1997YK55200001.

48. Glater EE, Rockman MV, Bargmann CI. Multigenic Natural Variation Underlies Caenorhabditis elegans Olfactory Preference for the Bacterial Pathogen Serratia marcescens. G3-Genes Genom Genet. 2014;4(2):265-76. doi: 10.1534/g3.113.008649.

PubMed PMID: WOS:000331614800007.

49. Gleason JM, Nuzhdin SV, Ritchie MG. Quantitative trait loci affecting a courtship signal in Drosophila melanogaster. Heredity. 2002;89:1-6. doi: 10.1038/sj.hdy.6800099. PubMed PMID: WOS:000176798300001.

50. Gleason JM, Ritchie MG. Do quantitative trait loci (QTL) for a courtship song difference between Drosophila simulans and D. sechellia coincide with candidate genes and intraspecific QTL? Genetics. 2004;166(3):1303-11. doi: DOI 10.1534/genetics.166.3.1303. PubMed PMID: WOS:000220950100017.

51. Greenwood AK, Ardekani R, McCann SR, Dubin ME, Sullivan A, Bensussen S, et al. Genetic mapping of natural variation in schooling tendency in the threespine stickleback. G3 (Bethesda). 2015;5(5):761-9. doi: 10.1534/g3.114.016519. PubMed PMID: 25717151; PubMed Central PMCID: PMCPMC4426364.

52. Greenwood AK, Wark AR, Yoshida K, Peichel CL. Genetic and neural modularity underlie the evolution of schooling behavior in threespine sticklebacks. Curr Biol. 2013;23(19):1884-8. doi: 10.1016/j.cub.2013.07.058. PubMed PMID: 24035541; PubMed Central PMCID: PMCPMC3828509.

53. Grisel JE, Belknap JK, OToole LA, Helms ML, Wenger CD, Crabbe JC. Quantitative trait loci affecting methamphetamine responses in BXD recombinant inbred mouse strains. Journal of Neuroscience. 1997;17(2):745-54. PubMed PMID: WOS:A1997WC27200023.

54. Gutierrez-Gil B, Ball N, Burton D, Haskell M, Williams JL, Wiener P. Identification of Quantitative Trait Loci Affecting Cattle Temperament. Journal of Heredity. 2008;99(6):629-38. doi: 10.1093/jhered/esn060. PubMed PMID:

WOS:000260152600010.

55. Ha JH, Alam M, Lee DH, Kim JJ. Whole Genome Association Study to Detect Single Nucleotide Polymorphisms for Behavior in Sapsaree Dog (Canis familiaris). Asian-Australas J Anim Sci. 2015;28(7):936-42. doi: 10.5713/ajas.14.0941. PubMed PMID: 26104397; PubMed Central PMCID: PMCPMC4478502.

56. Hain HS, Crabbe JC, Bergeson SE, Belknap JK. Cocaine-induced seizure thresholds: Quantitative trait loci detection and mapping in two populations derived from the C59BL/6 and DBA/2 mouse strains. Journal of Pharmacology and Experimental Therapeutics. 2000;293(1):180-7. PubMed PMID: WOS:000086664800023.

57. Harbison ST, McCoy LJ, Mackay TFC. Genome-wide association study of sleep in Drosophila melanogaster. Bmc Genomics. 2013;14. doi: Artn 28110.1186/1471-2164-14-281. PubMed PMID: WOS:000318525300001.

58. Hazard D, Moreno C, Foulquie D, Delval E, Francois D, Bouix J, et al. Identification of QTLs for behavioral reactivity to social separation and humans in sheep using the OvineSNP50 BeadChip. Bmc Genomics. 2014;15. doi: Artn 77810.1186/1471-2164-15-778. PubMed PMID: WOS:000341791100003.

59. Herrera VL, Pasion KA, Tan GA, Ruiz-Opazo N. Dahl (S x R) Rat Congenic Strain Analysis Confirms and Defines a Chromosome 17 Spatial Navigation Quantitative Trait Locus to < 10 Mbp. Plos One. 2013;8(2). doi: ARTN e5828010.1371/journal.pone.0058280. PubMed PMID: WOS:000315524900248.

60. Hiendleder S, Thomsen H, Reinsch N, Bennewitz J, Leyhe-Horn B, Looft C, et al. Mapping of QTL for body conformation and behavior in cattle. Journal of Heredity. 2003;94(6):496-506. doi: 10.1093/jhered/esg090. PubMed PMID:

WOS:000187987900008.

61. Hitzemann R, Cipp L, Demarest K, Mahjubi E, McCaughran J. Genetics of ethanol-induced locomotor activation: detection of QTLs in a C57BL/6JxDBA/2JF(2) intercross. Mammalian Genome. 1998;9(12):956-62. doi: DOI 10.1007/s003359900906. PubMed PMID: WOS:000077934900006.

62. Hitzemann R, Demarest K, Koyner J, Cipp L, Patel N, Rasmussen E, et al. Effect of genetic cross on the detection of quantitative trait loci and a novel approach to mapping QTLs. Pharmacol Biochem Be. 2000;67(4):767-72. doi: Doi 10.1016/S0091-3057(00)00421-4. PubMed PMID: WOS:000166747600012.

63. Hofstetter JR, Gifford KL, Trofatter JA, Mayeda AR. Altered circadian period of locomotor activity in carbonic anhydrase II-deficient mice. Biol Rhythm Res. 1999;30(5):517-28. doi: DOI 10.1076/brhm.30.5.517.1396. PubMed PMID: WOS:000084217800004.

64. Hofstetter JR, Mayeda AR. Provisional quantitative trait loci (QTL) for the Aschoff effect in RI mice. Physiology & Behavior. 1998;64(1):97-101. doi: Doi 10.1016/S0031-9384(98)00031-6. PubMed PMID: WOS:000074398100014.

65. Hosoya S, Suetake H, Suzuki Y, Kikuchi K. Genetic Basis Underlying Behavioral Correlation Between Fugu Takifugu rubripes and a Closely Related Species, Takifugu niphobles. Behavior Genetics. 2015;45(5):560-72. doi: 10.1007/s10519-015-9728-4. PubMed PMID: WOS:000360840800007.

66. Hunt GJ, Collins AM, Rivera R, Page RE, Guzman-Novoa E. Quantitative trait loci influencing honeybee alarm pheromone levels. Journal of Heredity. 1999;90(5):585-9. doi: DOI 10.1093/jhered/90.5.585. PubMed PMID: WOS:000083254100020.

67. Hunt GJ, Guzman-Novoa E, Fondrk MK, Page RE, Jr. Quantitative trait loci for honey bee stinging behavior and body size. Genetics. 1998;148(3):1203-13. PubMed PMID: 9539435; PubMed Central PMCID: PMCPMC1460054.

68. Hunt GJ, Page RE, Jr., Fondrk MK, Dullum CJ. Major quantitative trait loci affecting honey bee foraging behavior. Genetics. 1995;141(4):1537-45. PubMed PMID: 8601492; PubMed Central PMCID: PMCPMC1206885.

69. Huttunen S, Aspi J. Complex inheritance of male courtship song characters in Drosophila virilis. Behav Genet. 2003;33(1):17-24. PubMed PMID: 12645818.

70. Huttunen S, Aspi J. Complex inheritance of male courtship song characters in Drosophila virilis. Behavior Genetics. 2003;33(1):17-24. doi: Doi 10.1023/A:1021095331850. PubMed PMID: WOS:000179201400003.

71. Jones-Davis DM, Yang M, Rider E, Osbun NC, da Gente GJ, Li J, et al. Quantitative Trait Loci for Interhemispheric Commissure Development and Social Behaviors in the BTBR T+ tf/J Mouse Model of Autism. Plos One. 2013;8(4). doi: ARTN e61829 10.1371/journal.pone.0061829. PubMed PMID: WOS:000317563300039.

72. Jordan KW, Craver KL, Magwire MM, Cubilla CE, Mackay TF, Anholt RR. Genome-wide association for sensitivity to chronic oxidative stress in Drosophila melanogaster. Plos One. 2012;7(6):e38722. doi: 10.1371/journal.pone.0038722. PubMed PMID: 22715409; PubMed Central PMCID: PMCPMC3371005.

73. Joyner CP, Myrick LC, Crossland JP, Dawson WD. Deer Mice As Laboratory Animals. ILAR J. 1998;39(4):322-30. PubMed PMID: 11406688.

74. Kai W, Kikuchi K, Tohari S, Chew AK, Tay A, Fujiwara A, et al. Integration of the genetic map and genome assembly of fugu facilitates insights into distinct features of

genome evolution in teleosts and mammals. Genome Biol Evol. 2011;3:424-42. doi: 10.1093/gbe/evr041. PubMed PMID: 21551351.

75. Kirchmaier S, Naruse K, Wittbrodt J, Loosli F. The genomic and genetic toolbox of the teleost medaka (Oryzias latipes). Genetics. 2015;199(4):905-18. doi: 10.1534/genetics.114.173849. PubMed PMID: 25855651; PubMed Central PMCID: PMCPMC4391551.

76. Kitano J, Ross JA, Mori S, Kume M, Jones FC, Chan YF, et al. A role for a neo-sex chromosome in stickleback speciation. Nature. 2009;461(7267):1079-83. doi: 10.1038/nature08441. PubMed PMID: 19783981; PubMed Central PMCID: PMCPMC2776091.

77. Kowalko JE, Rohner N, Rompani SB, Peterson BK, Linden TA, Yoshizawa M, et al. Loss of Schooling Behavior in Cavefish through Sight-Dependent and Sight-Independent Mechanisms. Curr Biol. 2013;23(19):1874-83. doi: 10.1016/j.cub.2013.07.056. PubMed PMID: WOS:000326199700022.

78. Kupper C, Stocks M, Risse JE, Dos Remedios N, Farrell LL, McRae SB, et al. A supergene determines highly divergent male reproductive morphs in the ruff. Nat Genet. 2016;48(1):79-83. doi: 10.1038/ng.3443. PubMed PMID: 26569125.

79. Laine VN, Herczeg G, Shikano T, Vilkki J, Merila J. QTL analysis of behavior in nine-spined sticklebacks (Pungitius pungitius). Behav Genet. 2014;44(1):77-88. doi: 10.1007/s10519-013-9624-8. PubMed PMID: 24190427.

80. Laine VN, Shikano T, Herczeg G, Vilkki J, Merila J. Quantitative trait loci for growth and body size in the nine-spined stickleback Pungitius pungitius L. Molecular Ecology. 2013;22(23):5861-76. doi: 10.1111/mec.12526. PubMed PMID:

WOS:000327278700011.

81. Lamichhaney S, Fan GY, Widemo F, Gunnarsson U, Thalmann DS, Hoeppner MP, et al. Structural genomic changes underlie alternative reproductive strategies in the ruff (Philomachus pugnax). Nature Genetics. 2016;48(1):84-+. doi: 10.1038/ng.3430. PubMed PMID: WOS:000367255300018.

82. Le Roy I, Perez-Diaz F, Cherfouh A, Roubertoux PL. Preweanling sensorial and motor development in laboratory mice: Quantitative Trait Loci mapping. Developmental Psychobiology. 1999;34(2):139-58. doi: Doi 10.1002/(Sici)1098-2302(199903)34:2<139::Aid-Dev7>3.0.Co;2-H. PubMed PMID: WOS:000078995500007.

83. Li M, Tian S, Yeung CK, Meng X, Tang Q, Niu L, et al. Whole-genome sequencing of Berkshire (European native pig) provides insights into its origin and domestication. Sci Rep. 2014;4:4678. doi: 10.1038/srep04678. PubMed PMID: 24728479; PubMed Central PMCID: PMCPMC3985078.

84. Lightfoot JT, Turner MJ, Knab AK, Jedlicka AE, Oshimura T, Marzec J, et al. Quantitative trait loci associated with maximal exercise endurance in mice. J Appl Physiol. 2007;103(1):105-10. doi: 10.1152/japplphysiol.01328.2006. PubMed PMID: WOS:000248410900015.

85. Limousin D, Streiff R, Courtois B, Dupuy V, Alem S, Greenfield MD. Genetic architecture of sexual selection: QTL mapping of male song and female receiver traits in an acoustic moth. Plos One. 2012;7(9):e44554. doi: 10.1371/journal.pone.0044554. PubMed PMID: 22957082; PubMed Central PMCID: PMCPMC3434148.

86. Lu G, Bernatchez L. A study of fluctuating asymmetry in hybrids of dwarf and normal

lake whitefish ecotypes (Coregonus clupeaformis) from different glacial races. Heredity (Edinb). 1999;83 (Pt 6):742-7. PubMed PMID: 10651919.

87. Mackay TF, Richards S, Stone EA, Barbadilla A, Ayroles JF, Zhu D, et al. The Drosophila melanogaster Genetic Reference Panel. Nature. 2012;482(7384):173-8. doi: 10.1038/nature10811. PubMed PMID: 22318601; PubMed Central PMCID: PMCPMC3683990.

88. Markel PD, Bennett B, Beeson M, Gordon L, Johnson TE. Confirmation of quantitative trait loci for ethanol sensitivity in long-sleep and short-sleep mice. Genome Research. 1997;7(2):92-9. doi: DOI 10.1101/gr.7.2.92. PubMed PMID: WOS:A1997WH93600002.

89. Mayeda AR, Hofstetter JR. A QTL for the genetic variance in free-running period and level of locomotor activity between inbred strains of mice. Behavior Genetics. 1999;29(3):171-6. doi: Doi 10.1023/A:1021639901679. PubMed PMID: WOS:000083260600004.

90. Mcclearn GE, Kakihana R. Selective Breeding for Ethanol Sensitivity in Mice. Behavior Genetics. 1973;3(4):409-10. PubMed PMID: WOS:A1973R871300034.

91. McGrath PT, Rockman MV, Zimmer M, Jang H, Macosko EZ, Kruglyak L, et al. Quantitative Mapping of a Digenic Behavioral Trait Implicates Globin Variation in C. elegans Sensory Behaviors. Neuron. 2009;61(5):692-9. doi: 10.1016/j.neuron.2009.02.012. PubMed PMID: WOS:000264366900009.

92. Melo JA, Shendure J, Pociask K, Silver LM. Identification of sex specific quantitative trait loci controlling alcohol preference in C57BL/6 mice. Nature Genetics. 1996;13(2):147-53. doi: DOI 10.1038/ng0696-147. PubMed PMID:

WOS:A1996UN39500012.

93. Mendelson TC, Shaw KL. Sexual behaviour: rapid speciation in an arthropod. Nature. 2005;433(7024):375-6. doi: 10.1038/433375a. PubMed PMID: 15674280.

94. Minvielle F, Oguz Y. Effects of genetics and breeding on egg quality of Japanese quail. World Poultry Sci J. 2002;58(3):291-5. doi: Doi 10.1079/Wps20020022. PubMed PMID: WOS:000178138600003.

95. Mirol PM, Routtu J, Hoikkala A, Butlin RK. Signals of demographic expansion in Drosophila virilis. BMC Evol Biol. 2008;8:59. doi: 10.1186/1471-2148-8-59. PubMed PMID: 18298823; PubMed Central PMCID: PMCPMC2276204.

96. Moehring AJ, Llopart A, Elwyn S, Coyne JA, Mackay TFC. The genetic basis of prezygotic reproductive isolation between Drosophila santomea and D-yakuba due to mating preference. Genetics. 2006;173(1):215-23. doi: 10.1534/genetics.105.052993. PubMed PMID: WOS:000237990600018.

97. Moehring AJ, Mackay TFC. The quantitative genetic basis of male mating behavior in Drosophila melanogaster. Genetics. 2004;167(3):1249-63. doi: 10.1534/genetics.103.024372. PubMed PMID: WOS:000223109300019.

98. Moisan MP, Courvoisier H, Bihoreau MT, Gauguier D, Hendley ED, Lathrop M, et al. A major quantitative trait locus influences hyperactivity in the WKHA rat. Nature Genetics. 1996;14(4):471-3. doi: DOI 10.1038/ng1296-471. PubMed PMID: WOS:A1996VV73000026.

99. Morales-Hojas R, Reis M, Vieira CP, Vieira J. Resolving the phylogenetic relationships and evolutionary history of the Drosophila virilis group using multilocus data. Mol Phylogenet Evol. 2011;60(2):249-58. doi: 10.1016/j.ympev.2011.04.022.

PubMed PMID: 21571080.

100. Morozova TV, Huang W, Pray VA, Whitham T, Anholt RR, Mackay TF. Polymorphisms in early neurodevelopmental genes affect natural variation in alcohol sensitivity in adult drosophila. BMC Genomics. 2015;16:865. doi: 10.1186/s12864-015-2064-5. PubMed PMID: 26503115; PubMed Central PMCID: PMCPMC4624176.

101. Osborne KA, Robichon A, Burgess E, Butland S, Shaw RA, Coulthard A, et al. Natural behavior polymorphism due to a cGMP-dependent protein kinase of Drosophila. Science. 1997;277(5327):834-6. doi: DOI 10.1126/science.277.5327.834. PubMed PMID: WOS:A1997XQ24700049.

102. Owen EH, Christensen SC, Paylor R, Wehner JM. Identification of quantitative trait loci involved in contextual and auditory-cued fear conditioning in BXD recombinant inbred strains. Behav Neurosci. 1997;111(2):292-300. PubMed PMID: WOS:A1997WT65600006.

103. Page RE, Fondrk MK, Hunt GJ, Guzman-Novoa E, Humphries MA, Nguyen K, et al. Genetic dissection of honeybee (Apis mellifera L.) foraging behavior. Journal of Heredity. 2000;91(6):474-9. doi: DOI 10.1093/jhered/91.6.474. PubMed PMID: WOS:000166819000008.

104. Peccoud J, Ollivier A, Plantegenest M, Simon JC. A continuum of genetic divergence from sympatric host races to species in the pea aphid complex. Proc Natl Acad Sci U S A. 2009;106(18):7495-500. doi: 10.1073/pnas.0811117106. PubMed PMID: 19380742; PubMed Central PMCID: PMCPMC2678636.

105. Peirce JL, Derr R, Shendure J, Kolata T, Silver LM. A major influence of sex-specific

loci on alcohol preference in C57Bl/6 and DBA/2 inbred mice. Mammalian Genome. 1998;9(12):942-8. doi: DOI 10.1007/s003359900904. PubMed PMID: WOS:000077934900004.

106. Peripato AC, de Brito RA, Vaughn TT, Pletscher LS, Matioli SR, Cheverud JM. Quantitative trait loci for maternal performance for offspring survival in mice. Genetics. 2002;162(3):1341-53. PubMed PMID: WOS:000179739900029.

107. Phillips TJ, Belknap JK, Buck KJ, Cunningham CL. Genes on mouse Chromosomes 2 and 9 determine variation in ethanol consumption. Mammalian Genome. 1998;9(12):936-41. PubMed PMID: WOS:000077934900003.

108. Phillips TJ, Huson MG, McKinnon CS. Localization of genes mediating acute and sensitized locomotor responses to cocaine in BXD/Ty recombinant inbred mice. Journal of Neuroscience. 1998;18(8):3023-34. PubMed PMID: WOS:000072933300023.

109. Poissant J, Reale D, Martin JGA, Festa-Bianchet M, Coltman DW. A quantitative trait locus analysis of personality in wild bighorn sheep. Ecology and Evolution. 2013;3(3):474-81. doi: 10.1002/ece3.468. PubMed PMID: WOS:000316124100002.

110. Protas M, Jeffery WR. Evolution and development in cave animals: from fish to crustaceans. Wiley Interdiscip Rev Dev Biol. 2012;1(6):823-45. doi: 10.1002/wdev.61. PubMed PMID: 23580903; PubMed Central PMCID: PMCPMC3620605.

111. Prud'homme B, Gompel N, Rokas A, Kassner VA, Williams TM, Yeh SD, et al. Repeated morphological evolution through cis-regulatory changes in a pleiotropic gene. Nature. 2006;440(7087):1050-3. doi: 10.1038/nature04597. PubMed PMID:

16625197.

112. Purcell S, Neale B, Todd-Brown K, Thomas L, Ferreira MA, Bender D, et al. PLINK: a tool set for whole-genome association and population-based linkage analyses. Am J Hum Genet. 2007;81(3):559-75. doi: 10.1086/519795. PubMed PMID: 17701901; PubMed Central PMCID: PMCPMC1950838.

113. Radcliffe RA, Bohl ML, Lowe MV, Cycowski CS, Wehner JM. Mapping of quantitative trait loci for hypnotic sensitivity to ethanol in crosses derived from the C57BL/6 and DBA/2 mouse strains. Alcoholism-Clinical and Experimental Research. 2000;24(9):1335-42. doi: DOI 10.1111/j.1530-0277.2000.tb02101.x. PubMed PMID: WOS:000089364800003.

114. Ramos A, Moisan MP, Chaouloff F, Mormede C, Mormede P. Identification of female-specific QTLs affecting an emotionality-related behavior in rats. Mol Psychiatr. 1999;4(5):453-62. doi: DOI 10.1038/sj.mp.4000546. PubMed PMID: WOS:000083116200015.

115. Recoquillay J, Pitel F, Arnould C, Leroux S, Dehais P, Moreno C, et al. A medium density genetic map and QTL for behavioral and production traits in Japanese quail. BMC Genomics. 2015;16:10. doi: 10.1186/s12864-014-1210-9. PubMed PMID: 25609057; PubMed Central PMCID: PMCPMC4307178.

116. Reiner G, Kohler F, Berge T, Fischer R, Hubner-Weitz K, Scholl J, et al. Mapping of quantitative trait loci affecting behaviour in swine. Anim Genet. 2009;40(4):366-76. doi: 10.1111/j.1365-2052.2008.01847.x. PubMed PMID: 19291137.

117. Risinger FO, Quick E, Belknap JK. Quantitative trait loci for acute behavioral sensitivity to paraoxon. Neurotoxicol Teratol. 2000;22(5):667-74. doi: Doi

10.1016/S0892-0362(00)00085-4. PubMed PMID: WOS:000165769900005.

118. Rogers SM, Bernatchez L. The genetic architecture of ecological speciation and the association with signatures of selection in natural lake whitefish (Coregonus sp. Salmonidae) species pairs. Mol Biol Evol. 2007;24(6):1423-38. doi: 10.1093/molbev/msm066. PubMed PMID: 17404398.

119. Rueppell O, Pankiw T, Nielsen DI, Fondrk MK, Beye M, Page RE, Jr. The genetic architecture of the behavioral ontogeny of foraging in honeybee workers. Genetics. 2004;167(4):1767-79. doi: 10.1534/genetics.103.021949. PubMed PMID: 15342515; PubMed Central PMCID: PMCPMC1471018.

120. Sauce B, de Brito RA, Peripato AC. Genetic architecture of nest building in mice LG/J x SM/J. Front Genet. 2012;3:90. doi: 10.3389/fgene.2012.00090. PubMed PMID: 22654894; PubMed Central PMCID: PMCPMC3361010.

121. Schutz K, Kerje S, Carlborg O, Jacobsson L, Andersson L, Jensen P. QTL analysis of a red junglefowl x White Leghorn intercross reveals trade-off in resource allocation between behavior and production traits. Behav Genet. 2002;32(6):423-33. PubMed PMID: 12467340.

122. Seltzer Z, Wu TX, Max MB, Diehl SR. Mapping a gene for neuropathic pain-related behavior following peripheral neurectomy in the mouse. Pain. 2001;93(2):101-6. doi: Doi 10.1016/S0304-3959(01)00295-0. PubMed PMID: WOS:000169804600002.

123. Shapiro MD, Bell MA, Kingsley DM. Parallel genetic origins of pelvic reduction in vertebrates. Proc Natl Acad Sci U S A. 2006;103(37):13753-8. doi: 10.1073/pnas.0604706103. PubMed PMID: 16945911; PubMed Central PMCID: PMCPMC1564237.

124. Shaw KL, Lesnick SC. Genomic linkage of male song and female acoustic preference QTL underlying a rapid species radiation. P Natl Acad Sci USA. 2009;106(24):9737-42. doi: 10.1073/pnas.0900229106. PubMed PMID: WOS:000267045500036.

125. Shaw KL, Parsons YM, Lesnick SC. QTL analysis of a rapidly evolving speciation phenotype in the Hawaiian cricket Laupala. Molecular Ecology. 2007;16(14):2879-92. doi: 10.1111/j.1365-294X.2007.03321.x. PubMed PMID: WOS:000247757200009.

126. Shikano T, Shimada Y, Herczeg G, Merila J. History vs. habitat type: explaining the genetic structure of European nine-spined stickleback (Pungitius pungitius) populations. Mol Ecol. 2010;19(6):1147-61. doi: 10.1111/j.1365-294X.2010.04553.x. PubMed PMID: 20163545.

127. Shimomura K, Low-Zeddies SS, King DP, Steeves TDL, Whiteley A, Kushla J, et al. Genome-wide epistatic interaction analysis reveals complex genetic determinants of circadian behavior in mice. Genome Research. 2001;11(6):959-80. doi: DOI 10.1101/gr.171601. PubMed PMID: WOS:000169045600006.

128. Shorter J, Couch C, Huang W, Carbone MA, Peiffer J, Anholt RR, et al. Genetic architecture of natural variation in Drosophila melanogaster aggressive behavior. Proc Natl Acad Sci U S A. 2015;112(27):E3555-63. doi: 10.1073/pnas.1510104112. PubMed PMID: 26100892; PubMed Central PMCID: PMCPMC4500262.

129. Shorter JR, Arechavaleta-Velasco M, Robles-Rios C, Hunt GJ. A Genetic Analysis of the Stinging and Guarding Behaviors of the Honey Bee. Behavior Genetics. 2012;42(4):663-74. doi: 10.1007/s10519-012-9530-5. PubMed PMID: WOS:000305232000013.

130. Sokoloff G, Parker CC, Lim JE, Palmer AA. Anxiety and fear in a cross of C57BL/6J

and DBA/2J mice: mapping overlapping and independent QTL for related traits. Genes Brain Behav. 2011;10(5):604-14. doi: 10.1111/j.1601-183X.2011.00699.x. PubMed PMID: WOS:000292456900012.

131. Storey AA, Athens JS, Bryant D, Carson M, Emery K, deFrance S, et al. Investigating the global dispersal of chickens in prehistory using ancient mitochondrial DNA signatures. Plos One. 2012;7(7):e39171. doi: 10.1371/journal.pone.0039171. PubMed PMID: 22848352; PubMed Central PMCID: PMCPMC3405094.

132. Suzuki T, Ishikawa A, Nishimura M, Yoshimura T, Namikawa T, Ebihara S. Mapping quantitative trait loci for circadian behavioral rhythms in SMXA recombinant inbred strains. Behavior Genetics. 2000;30(6):447-53. doi: Doi 10.1023/A:1010298701251. PubMed PMID: WOS:000170469700003.

133. Swarup S, Huang W, Mackay TF, Anholt RR. Analysis of natural variation reveals neurogenetic networks for Drosophila olfactory behavior. Proc Natl Acad Sci U S A. 2013;110(3):1017-22. doi: 10.1073/pnas.1220168110. PubMed PMID: 23277560; PubMed Central PMCID: PMCPMC3549129.

134. Tarantino LM, McClearn GE, Rodriguez LA, Plomin R. Confirmation of quantitative trait loci for alcohol preference in mice. Alcoholism-Clinical and Experimental Research. 1998;22(5):1099-105. PubMed PMID: WOS:000075475100018.

135. Tschinkel WR. Fire ant queen longevity and age: estimation by sperm depletion. Ann Entomol Soc Am. 1987;80:263-6.

136. Tsuboko S, Kimura T, Shinya M, Suehiro Y, Okuyama T, Shimada A, et al. Genetic Control of Startle Behavior in Medaka Fish. Plos One. 2014;9(11). doi: ARTN e11252710.1371/journal.pone.0112527. PubMed PMID: WOS:000347709300064.

137. Tsuruda JM, Harris JW, Bourgeois L, Danka RG, Hunt GJ. High-resolution linkage analyses to identify genes that influence Varroa sensitive hygiene behavior in honey bees. Plos One. 2012;7(11):e48276. doi: 10.1371/journal.pone.0048276. PubMed PMID: 23133626; PubMed Central PMCID: PMCPMC3487727.

138. Turri MG, Datta SR, DeFries J, Henderson ND, Flint J. QTL analysis identifies multiple behavioral dimensions in ethological tests of anxiety in laboratory mice. Curr Biol. 2001;11(10):725-34. doi: Doi 10.1016/S0960-9822(01)00206-8. PubMed PMID: WOS:000168765100014.

139. Turri MG, Henderson ND, DeFries JC, Flint J. Quantitative trait locus mapping in laboratory mice derived from a replicated selection experiment for open-field activity. Genetics. 2001;158(3):1217-26. PubMed PMID: WOS:000169936600026.

140. Valentinuzzi VS, Kolker DE, Vitaterna MH, Shimomura K, Whiteley A, Low-Zeddies S, et al. Automated measurement of mouse freezing behavior and its use for quantitative trait locus analysis of contextual fear conditioning in (BALB/cJ x C57BL/6J)F-2 mice. Learn Memory. 1998;5(4-5):391-403. PubMed PMID: WOS:000076812700012.

141. Verkuil YI, Juillet C, Lank DB, Widemo F, Piersma T. Genetic variation in nuclear and mitochondrial markers supports a large sex difference in lifetime reproductive skew in a lekking species. Ecol Evol. 2014;4(18):3626-32. doi: 10.1002/ece3.1188. PubMed PMID: 25478153; PubMed Central PMCID: PMCPMC4224536.

142. Vonesch SC, Lamparter D, Mackay TF, Bergmann S, Hafen E. Genome-Wide Analysis Reveals Novel Regulators of Growth in Drosophila melanogaster. PLoS Genet. 2016;12(1):e1005616. doi: 10.1371/journal.pgen.1005616. PubMed PMID:

26751788; PubMed Central PMCID: PMCPMC4709145.

143. Wallberg A, Han F, Wellhagen G, Dahle B, Kawata M, Haddad N, et al. A worldwide survey of genome sequence variation provides insight into the evolutionary history of the honeybee Apis mellifera. Nat Genet. 2014;46(10):1081-8. doi: 10.1038/ng.3077. PubMed PMID: 25151355.

144. Wang J, Wurm Y, Nipitwattanaphon M, Riba-Grognuz O, Huang YC, Shoemaker D, et al. A Y-like social chromosome causes alternative colony organization in fire ants. Nature. 2013;493(7434):664-8. doi: 10.1038/nature11832. PubMed PMID: 23334415.

145. Wehner JM, Radcliffe RA, Rosmann ST, Christensen SC, Rasmussen DL, Fulker DW, et al. Quantitative trait locus analysis of contextual fear conditioning in mice. Nature Genetics. 1997;17(3):331-4. doi: DOI 10.1038/ng1197-331. PubMed PMID: WOS:A1997YD66700030.

146. Werren JH, Loehlin DW. The parasitoid wasp Nasonia: an emerging model system with haploid male genetics. Cold Spring Harb Protoc. 2009;2009(10):pdb emo134. doi: 10.1101/pdb.emo134. PubMed PMID: 20147035; PubMed Central PMCID: PMCPMC2916733.

147. Werren JH, Richards S, Desjardins CA, Niehuis O, Gadau J, Colbourne JK, et al. Functional and evolutionary insights from the genomes of three parasitoid Nasonia species. Science. 2010;327(5963):343-8. doi: 10.1126/science.1178028. PubMed PMID: 20075255; PubMed Central PMCID: PMCPMC2849982.

148. Whiteley AR, Bhat A, Martins EP, Mayden RL, Arunachalam M, Uusi-Heikkila S, et al. Population genomics of wild and laboratory zebrafish (Danio rerio). Mol Ecol. 2011;20(20):4259-76. doi: 10.1111/j.1365-294X.2011.05272.x. PubMed PMID:

21923777; PubMed Central PMCID: PMCPMC3627301.

149. Williams EG, Mouchiroud L, Frochaux M, Pandey A, Andreux PA, Deplancke B, et al. An Evolutionarily Conserved Role for the Aryl Hydrocarbon Receptor in the Regulation of Movement. Plos Genetics. 2014;10(9). doi: ARTN e100467310.1371/journal.pgen.1004673. PubMed PMID: WOS:000343009600062.

150. Wright D, Nakamichi R, Krause J, Butlin RK. QTL analysis of behavioral and morphological differentiation between wild and laboratory zebrafish (Danio rerio). Behavior Genetics. 2006;36(2):271-84. doi: 10.1007/s10519-005-9029-4. PubMed PMID: WOS:000237191600011.

151. Yamanoue Y, Miya M, Matsuura K, Katoh M, Sakai H, Nishida M. A new perspective on phylogeny and evolution of tetraodontiform fishes (Pisces : Acanthopterygii) based on whole mitochondrial genome sequences: Basal ecological diversification? Bmc Evolutionary Biology. 2008;8. doi: Artn 21210.1186/1471-2148-8-212. PubMed PMID: WOS:000258231100001.

152. Yang J, Lee SH, Goddard ME, Visscher PM. GCTA: a tool for genome-wide complex trait analysis. Am J Hum Genet. 2011;88(1):76-82. doi: 10.1016/j.ajhg.2010.11.011. PubMed PMID: 21167468; PubMed Central PMCID: PMCPMC3014363.

153. Yeh SD, True JR. The Genetic Architecture of Coordinately Evolving Male Wing Pigmentation and Courtship Behavior in Drosophila elegans and Drosophila gunungcola. G3-Genes Genom Genet. 2014;4(11):2079-93. doi: 10.1534/g3.114.013037. PubMed PMID: WOS:000345288700003.

154. Yoshikawa T, Watanabe A, Ishitsuka Y, Nakaya A, Nakatani N. Identification of multiple genetic loci linked to the propensity for "behavioral despair" in mice.

Genome Research. 2002;12(3):357-66. doi: 10.1101/gr/222602. PubMed PMID: WOS:000174171300001.

155. Yoshizawa M, Robinson BG, Duboue ER, Masek P, Jaggard JB, O'Quin KE, et al. Distinct genetic architecture underlies the emergence of sleep loss and prey-seeking behavior in the Mexican cavefish. Bmc Biol. 2015;13. doi: ARTN 1510.1186/s12915-015-0119-3. PubMed PMID: WOS:000351130300001.
